# PolNet: A Tool to Quantify Network-Level Cell Polarity and Blood Flow in Vascular Remodeling

**DOI:** 10.1016/j.bpj.2018.03.032

**Published:** 2018-05-08

**Authors:** Miguel O. Bernabeu, Martin L. Jones, Rupert W. Nash, Anna Pezzarossa, Peter V. Coveney, Holger Gerhardt, Claudio A. Franco

**Affiliations:** 1 Centre for Medical Informatics, Usher Institute, The University of Edinburgh, Edinburgh, United Kingdom; 2 Centre for Computational Science, Department of Chemistry, University College London, London, United Kingdom; 3 Electron Microscopy Science Technology Platform, The Francis Crick Institute, London, United Kingdom; 4 EPCC, School of Physics and Astronomy, The University of Edinburgh, Edinburgh, United Kingdom; 5 Instituto de Medicina Molecular, Faculdade de Medicina, Universidade de Lisboa, Lisboa, Portugal; 6 Max Delbrück Center for Molecular Medicine in the Helmholtz Association, Berlin, Germany; 7 Vascular Patterning Laboratory, Center for Cancer Biology, VIB, Leuven, Belgium; 8 Vascular Patterning Laboratory, Department of Oncology, KU Leuven, Leuven, Belgium; 9 German Center for Cardiovascular Research, Berlin, Germany; 10 Berlin Institute of Health, Berlin, Germany

## Abstract

In this article, we present PolNet, an open-source software tool for the study of blood flow and cell-level biological activity during vessel morphogenesis. We provide an image acquisition, segmentation, and analysis protocol to quantify endothelial cell polarity in entire in vivo vascular networks. In combination, we use computational fluid dynamics to characterize the hemodynamics of the vascular networks under study. The tool enables, to our knowledge for the first time, a network-level analysis of polarity and flow for individual endothelial cells. To date, PolNet has proven invaluable for the study of endothelial cell polarization and migration during vascular patterning, as demonstrated by two recent publications. Additionally, the tool can be easily extended to correlate blood flow with other experimental observations at the cellular/molecular level. We release the source code of our tool under the Lesser General Public License.

## Introduction

The establishment of a functional, patterned vascular network is crucial for development, tissue growth, and physiology. Conversely, mispatterning of vascular networks contributes to the pathogenesis of several diseases, including arteriovenous malformations, hemangioma, hereditary hemorrhagic telangiectasias, or venous cavernomas. Sprouting angiogenesis is one of the mechanisms responsible for expanding vascular networks into avascular areas. This process only generates a dense and immature network of vessels that requires subsequent extensive remodeling to form a functional, hierarchically branched network—a process termed vascular remodeling (1). In contrast to sprouting, the molecular mechanisms regulating vascular remodeling are poorly understood. The forces exerted by blood on the luminal surface of the endothelium (most notably wall shear stress (WSS)) have been recognized as primary drivers regulating vascular remodeling (2, 3, 4). Recent work by the authors and others identified that endothelial WSS regulates endothelial cell polarity and cell migration, orchestrating vascular remodeling (5, 6, 7, 8) and controlling vessel diameter (9, 10, 11, 12). Thus, there is increasing interest in understanding mechanistically how hemodynamic forces impact endothelial response at the cellular and molecular levels.

The mouse retina is one of the most popular models to use when investigating the molecular mechanisms governing angiogenesis. However, the way in which blood flow influences the development of the retinal vasculature remains elusive. Progress is hampered by the limitations of current assays used to probe the relationship between hemodynamics and molecular response and the complexity of in vivo vascular connectivity. To date, researchers have primarily used in vitro microfluidic assays to study the impact of blood flow on endothelial cell biology, extrapolating these observations to explain phenotypic changes in mouse mutants. Even though some authors have been able to measure microvascular WSS in vivo (see Lipowsky et al. (13) for a survey), this has never been achieved, to the best of our knowledge, in the context of vascular morphogenesis in the mouse retina model.

Our recent work on vascular remodeling established a strong connection between blood flow and endothelial cell polarity. We developed PolNet to be able to quantify the relationship between endothelial cell polarity and WSS. PolNet’s image-processing algorithms and computational fluid dynamics (CFD) simulator were described and validated by Bernabeu et al (14). PolNet was then successfully used in two recent publications: 1) in Franco et al. (5), we showed that flow-induced cell polarization directs migration of endothelial cells away from low-flow segments, and 2) in Franco et al. (6), we showed that noncanonical Wnt signaling modulates the endothelial shear stress flow sensor during vascular remodeling. In this article, we focus on providing a step-by-step description of our experimental and computational protocols to facilitate the adoption of our polarity-WSS analysis. Furthermore, we provide a Docker-based installation of our tool and release its source code under the open-source Lesser General Public License.

### Methods

The design and implementation of PolNet is better understood in the context of the complete protocol, comprising experimental and computational parts, used in Franco et al (5, 6). The materials and setup are described in Supporting Materials and Methods, Section A, and a step-by-step protocol is provided in Supporting Materials and Methods, Section B. Briefly, samples of murine retinal plexus are collected at different postnatal (P) days, fixed, and labeled for the luminal surface (ICAM2), cell nuclei (Erg), and Golgi apparatus (Golph4). The retinal vascular networks are imaged and postprocessed to generate a binary mask from the ICAM2 channel and a second image with at least the Erg and Golph4 channels. These two images define the input to PolNet. An example data set is provided in Supporting Materials and Methods, Section C. Based on this, PolNet can be used to quantify the relationship between endothelial cell polarity (defined for each cell as the vector p originating from the center of mass of the nucleus and directed to the center of mass of the Golgi complex) and the direction and magnitude of the computed traction vector t (i.e., the product of the deviatoric stress tensor and the surface normal), which we will refer to as the WSS vector, for convenience. PolNet provides a graphical user interface for the user to perform the following three tasks: 1) to construct a flow model from the ICAM2 mask and use the HemeLB flow solver (15) to estimate the WSS across the whole network (as well as blood velocity, shear rate, and pressure), 2) to interactively delineate the cell polarity vectors for each endothelial cell in the plexus based on the Erg-Golph4 image, and 3) to statistically analyze the relationship between the cell polarity and WSS vectors, **p** and **t**, respectively. PolNet currently offers the following analyses:

1) A directionality table shows the number and ratio of endothelial cells polarized against the flow direction.
2) A polar histogram shows the distribution of endothelial cell polarization angles in relation to the blood flow direction. Each plexus can also be subdivided into regions (e.g., artery, vein, capillary, and sprouting front), and each cell will be assigned to one of these vascular beds for further analysis. Multiple angular distributions can be subsequently compared using the Kuiper test to evaluate the likelihood that the two samples are drawn from the same underlying angular distribution.
3) The WSS sensor analysis allows us to bin the angular data according to the WSS magnitude to plot the proportion of cells oriented against the flow, which we define as having an angle of $180^{\circ }\pm 45^{\circ }$ with regard to the flow direction. We consider the threshold for polarization as the WSS value, leading to 60% of the endothelial cells being aligned against the flow direction.
4) Scalar product slope: we calculate the scalar product of the polarity and WSS vectors given by ‖p‖‖t‖cos(θ), where *θ* is the angle between them, which combines information about the length and relative angles of the vectors. By plotting the scalar product against ‖t‖, we can study and compare the plot slope, i.e., ‖p‖cos(θ), between groups. A larger negative slope corresponds to a larger polarization effect for a given WSS, which is a surrogate measure for the sensitivity of cells to flow.

#### PolNet software repositories and contributions

Users interested in the software package in its current state (with just the functionality described in this article) are advised to follow the installation instructions in Supporting Materials and Methods, Section A, on how to install and run the current version of PolNet through the Docker platform. For advanced users or tool developers who wish to extend/adapt the software, PolNet can be easily modified to perform similar analyses involving the comparison of in silico flow estimates and other experimental observations at the cellular/molecular level. Table 1 summarizes the software repositories hosting the different components of PolNet.

**Table 1:** Software Repositories Hosting the Code Used to Build the PolNet Tool

PolNet Component	Description	Software Repository
PolNet main	graphical user interface and main pre- and postprocessing algorithms	https://github.com/mobernabeu/polnet
PolNet Dockerfile	Docker-specific instructions to create the PolNet container	https://github.com/mobernabeu/docker-polnet
HemeLB	flow model setup tool and CFD solver	https://github.com/UCL/hemelb

Users interested in expanding the functionality of PolNet should obtain the code in the PolNet main repository and follow the installation instructions for developers. This will provide access to the source MATLAB (The MathWorks, Natick, MA) and Python scripts defining the graphical user interface and the main pre- and postprocessing algorithms. Users can then adapt the code to meet the requirements of their analyses and potentially contribute the changes back to the main repository. The user can build his/her own Docker container based on the instructions provided in our PolNet Dockerfile repository. Note that to run the HemeLB setup tool or solver as part of the PolNet developer version, a HemeLB developer installation is required. Please refer to the HemeLB repository for installation instructions. Similarly, users can report bugs and suggest new features to the PolNet developers (and other interested users) by creating new issues on the relevant GitHub repository.

#### Limitations of the method

*Capillary network requirements*. We employ the maximal intensity *z*-projection of a confocal microscopy image stack to segment the luminal space defining the flow domain. This implies that all the information in the *z* axis is projected onto a plane, and therefore, the method is only applicable to capillary beds where all the vessels can be considered coplanar in this projection. In the case of the neonatal mouse retina, this condition is fulfilled before P7, where a single nonoverlapping plexus covers the outer layers of the retina. If this condition is not fulfilled, vessels overlapping or going past each other at different depths will appear connected in the plexus segmentation, which will lead to inaccuracies in the simulated hemodynamics. This situation can be easily diagnosed if the image segmentation displays branching points with four afferent/efferent segments. Thus, this protocol is not suitable for mouse retinas beyond P7 or other vascular plexuses with complex three-dimensional (3D) organization. This would require segmentation of 3D image stacks, which is an aspect not yet implemented in the current version of PolNet. In addition, the method requires well-defined inlets (arteries) and outlets (veins) in the network for correct flow modeling. In the mouse retina, arteries and veins only become apparent after P3; thus, earlier stages cannot be reliably analyzed with PolNet. Special care also needs to be taken when analyzing mouse mutants strongly affecting vessel architecture. We advise against using PolNet in phenotypes such as 1) compromised arteries and/or venous differentiation, which lack clearly defined inlets/outlets; 2) dramatically affected vascular networks, including extremely overgrown capillary networks (e.g., Notch loss-of-function phenotypes (16)); and 3) artery-vein crossing phenotypes (e.g., Neuropillin(cyto)(Δ) retina (17)).

*Polarity delineation*. Assigning each Golgi apparatus to the corresponding endothelial nucleus is a manual operation and thus subject to human error. For accurate delineation, we recommend a computer setup with 1 or 2 monitors simultaneously displaying 1) the PolNet application for the nucleus and Golgi apparatus assignments and 2) an image analysis software (e.g., FIJI) to visualize each *z*-section of the *z*-stack and to turn on or off the display of the various image channels with the different stainings. The simultaneous visualization of the PolNet interface and the image of each *z*-section allows us to discriminate and pair each Golgi and nucleus, even in crowded regions. To minimize human error, we suggest that acquisition and polarity delineation are conducted by different persons. To blind the identity of the data set to the person delineating polarity vectors, abstract names should be given to each file.

*Accuracy of the flow estimates generated*. The accuracy of the WSS estimates produced by the flow solver is of paramount importance for the usefulness of PolNet. Here, we discuss some potential sources of error.

1) Vessel geometry reconstruction: the geometry of the flow domain has a strong influence on the computed hemodynamics. Therefore, it is critical that the process of plexus sample preparation for imaging is done with great care to avoid introducing artifacts (e.g., artificial vessel disconnections) and to ensure that the labeling and mounting protocol does not lead to significant shrinkage of the luminal space. Furthermore, the vessel reconstruction assumes a circular cross section. All these potential sources of error lead to uncertainty in the flow estimates, thus requiring quantification. The user can do this by comparing the flow results against existing experimental measurements of blood velocity in the mouse retinal vasculature (see Bernabeu et al. (14) and Table 2 for values compiled from the literature).

**Table 2:** WSS Values Reported in the Literature

Reference	Species, Tissue	Vessel	WSS (Pa)
(21)	cat, sartorius muscle	arterioles	6–14
		capillaries	1.2
		venules	0.3–1
(22)	human, various tissues	arteries	1–7
		veins	0.1–0.6
(23)	mouse, infrarenal aorta	artery	8.76±0.83
	rat, infrarenal aorta	artery	7.05±0.67
	human, infrarenal aorta	artery	0.48±0.03
2) Choice of inlet and outlet boundary conditions: the samples under study typically comprise only a subset of the retinal vascular plexus. These take the form of wedges with an artery that runs radially from the optic disc across the wedge and feeds capillary beds located at either side of it, which in turn are drained by veins that return in the same radial fashion to the optic disc (see Franco et al. (5, 6)). We term this as the vein-artery-vein (V-A-V) configuration, although other configurations are also possible (e.g., A-V and A-V-A). In addition to imposing no-slip boundary conditions at the vessel walls, we need to specify boundary conditions at the network inlets and outlets. In Bernabeu et al. (14), we surveyed the literature for experimental measurements of blood flow rate or pressure in the central retinal artery/vein to be used as boundary conditions. We concluded that the V-A-V configuration combined with pressure-boundary conditions minimizes the modeling error appearing in the capillary beds defined between each artery-vein pair (including the sprouting front) as well as in the arteries. These are our main regions of interest. Because of the lack of such measurements in the neonatal mouse, we used values measured in adult mice. This design decision can lead to inaccuracies in the flow estimates generated, which are difficult to quantify a priori. In Bernabeu et al. (14), we performed a sensitivity analysis of the inlet/outlet pressures used in our simulations and observed that, although velocity and WSS go up as the pressure difference between inlets and outlets increases, the main perfusion patterns (e.g., areas of relatively low/high flow, flow direction) remain unchanged for a wide range of inlet/outlet configurations and that the relative differences in WSS that have been shown to drive remodeling (5) are also preserved.
3) Choice of rheology model: blood is a dense suspension of deformable cells in blood plasma that leads to a complex range of rheological properties appearing at different scales. When using a CFD approach to simulate blood flow, one has to choose an appropriate approximation for this rheology. In this work, we use the homogeneous shear-thinning rheology model proposed in (14). This approach is a compromise between accuracy and computational tractability (18). However, it fails to capture certain hemorheological features (e.g., the plasma skimming and the Fåhræus-Lindqvist effects) when applied to the simulation of blood flow in small capillaries, especially as vessel caliber gets closer to or even smaller than the typical red blood cell diameter of ∼8μm.

### Results

In this section, we present an example of PolNet analysis performed on the data in Supporting Materials and Methods, Section C, of this article. We choose a subset of a P6 mouse retinal plexus for demonstration purposes. The interested reader can refer to (5, 6, 14) for analyses based on larger plexuses. Supporting Materials and Methods, Section D, provides troubleshooting advice.

**Figure 1:**
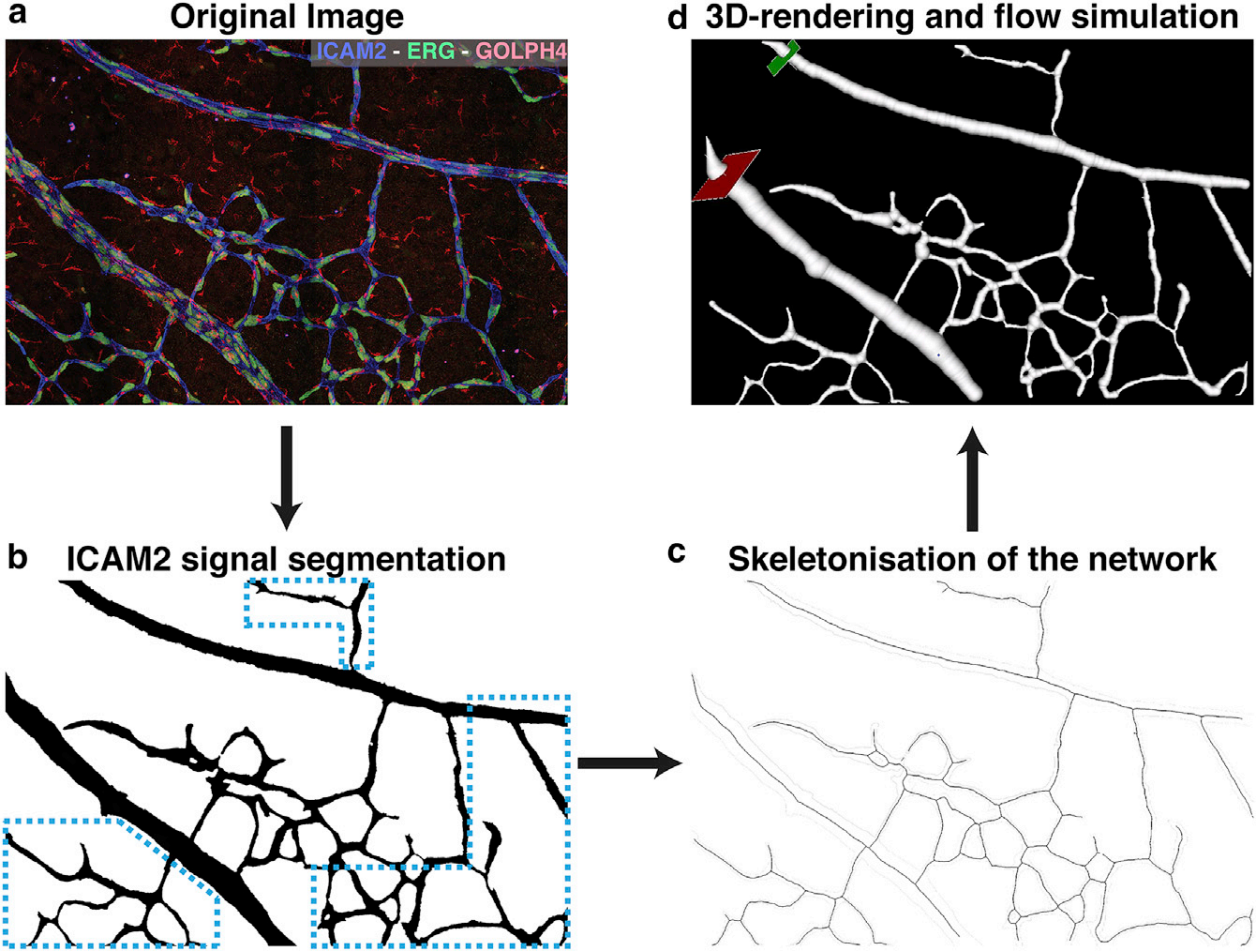
(*a*) Example of Golph4 (*red*, Golgi), Erg (*green*, nucleus), and ICAM2 (*blue*, luminal surface) stainings of a subset of a P6 mouse retinal plexus. (*b*) A binary mask generated from the ICAM2 staining in (*a*) is shown. Note that the hemodynamics recovered in the blue highlighted region will not be accurate because of the missing connections to the nearby vessels. The interested reader can refer to (5, 6, 14) for analyses based on larger plexuses. (*c*) Results of the skeletonisation step are shown. (*d*) A flow simulation setup defines the inlet, outlet, and seed position.

A P6 mouse retinal plexus was prepared, imaged, and postprocessed based on the instructions in Supporting Materials and Methods, Section B, steps 1–20. A subset of interest, including arteries, veins, and capillaries, was identified in the resulting images and cropped to simplify further analysis. Fig. 1 *a* shows the first output image combining the specific stainings (Golph4, Erg, and ICAM2). Fig. 1 *b* shows the second one with the segmentation of the ICAM2 channel.

The ICAM2 mask was processed with PolNet to construct a flow model. Fig. 1 *c* shows the results of the skeletonization step. Fig. S3 *a* presents the vessel caliber histogram generated by the surface reconstruction stage. Fig. 1 *d* shows a screenshot of the flow simulation setup operation (note the location of the flow inlet in *green* and flow outlet in *red*). Finally, Fig. S3 *b* presents the WSS histogram generated at the end of the simulation. Table 2 summarizes experimentally measured WSS values for simulation validation purposes.

**Figure 2:**
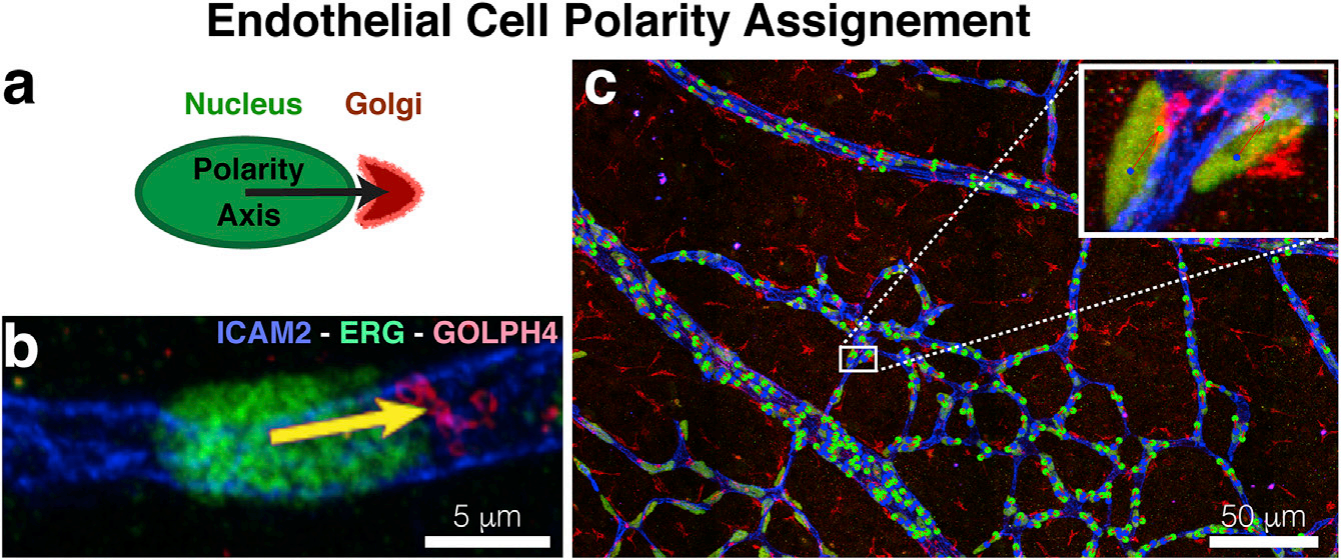
(*a*) Polarity vectors are defined between the approximate centers of mass of the nucleus and the Golgi of any given cell. (*b*) An example of a polarity vector on Golph4-Erg-ICAM2 stained plexus is shown. (*c*) A plexus-wide view of the polarity vector delineation on Fig. 1*a*. A close-in panel provides a detailed view of the polarity delineation on a subset of endothelial cells.

The Golph4-Erg-ICAM2 image was used to delineate cell polarity across the whole plexus with PolNet. This is achieved by placing, on top of the image being displayed, consecutive pairs of points corresponding to the approximate center of mass of the nucleus and Golgi of any given cell. Every time a new pair is added, an arrow connecting them is automatically drawn. This arrow defines the polarity vector of a given cell (see Fig. 2, *a* and *b*). Fig. 2 *c* shows the delineation result for all the cells of interest in our example data set.

**Figure 3:**
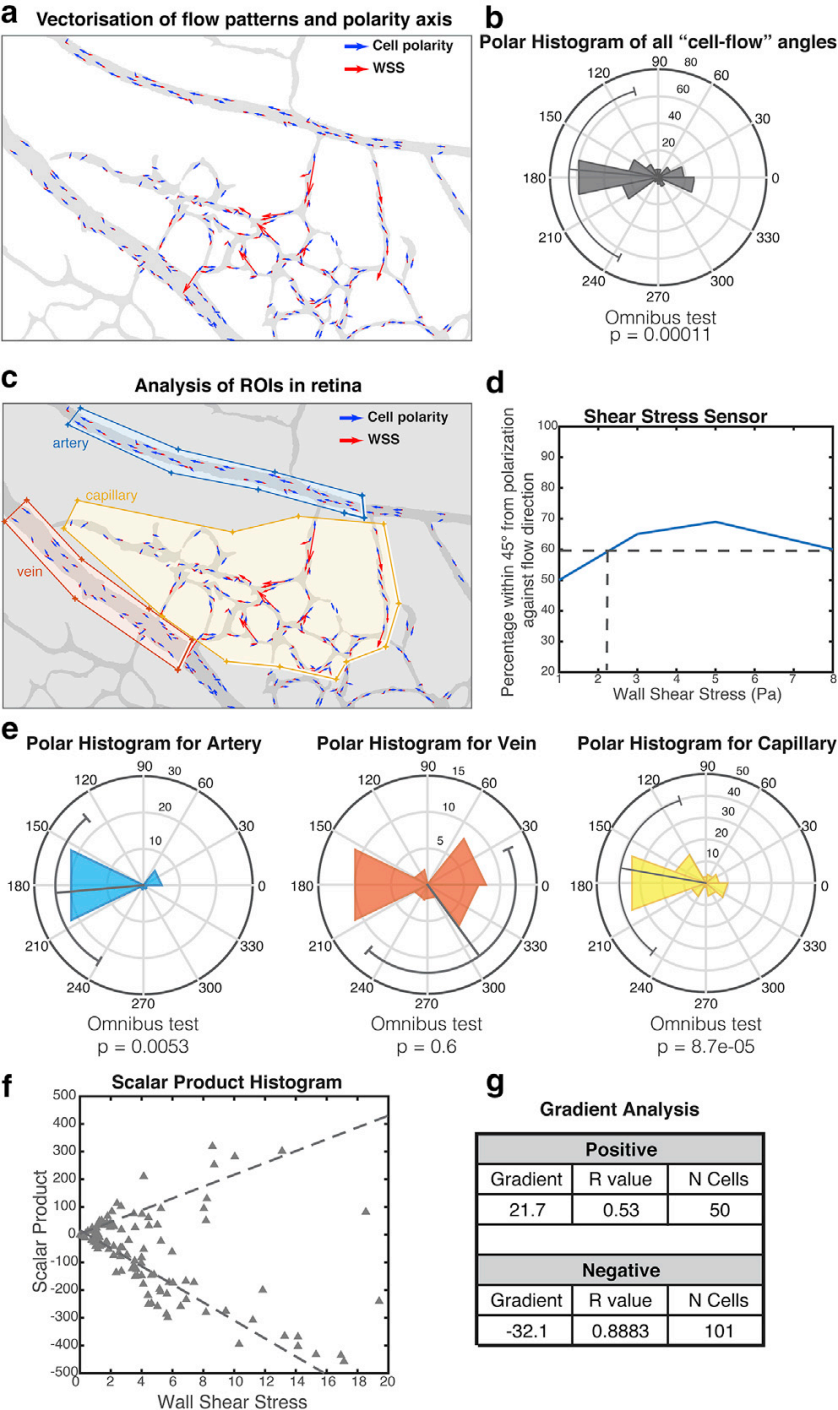
Polarity versus wall shear stress (WSS) analysis. (*a*) An overlay of a luminal mask (*gray*), polarity vectors (*blue*), and WSS vectors scaled according to magnitude (*red*) are shown. (*b*) A polar histogram with relative angles formed by the polarity and WSS vectors on each cell are shown, along with the results of a statistical test for random distribution. (*c*) An example of region subdivision is shown: yellow, orange, and blue polygons classify the endothelial cells into arterial, capillar, and venous, respectively. (*d*) Shear stress sensitivity analysis: the percentage of cells polarized against the flow (within 45° tolerance) is shown as a function of the WSS experienced. (*e*) The results of the polarity analysis are applied to each delineated region separately. (*f*) A scatterplot showing the scalar product value for every pair of WSS and polarity vector. (*g*) Scalar product quantification for both the positive and negative subgroups is shown: the gradient of linear regression, Pearson *r*-value, and number of cells.

Fig. 3 presents the results of the PolNet analysis on the example data set. Fig. 3 *a* shows the overlay of the luminal mask and the cell polarity vectors in blue and the flow vectors (WSS selected on the corresponding dropdown menu, not shown) in red. Fig. 3 *b* presents the quantification of the relative angles defined by each pair of polarity and flow vectors. It also includes the results of a statistical test for randomness in the distribution of these angles. Fig. 3 *c* displays an example of subdivision into three regions (arterial, venous, and capillary) for further analysis. Fig. 3 *d* presents the results of the WSS sensor analysis. Finally, Fig. 3 *e* shows the results of the polarity analysis applied to each of the individually selected regions.

### Discussion

PolNet was conceived to be an easily extensible tool for network-level quantitative research in vascular morphogenesis. To date, PolNet has proven invaluable for the study of endothelial cell polarization and migration during vascular patterning, as demonstrated by our recent articles (5, 6). We have taken special care to make the pipeline easily deployable across a broad range of computer configurations for easy adoption of the PolNet software by the scientific community. The workflow described in this article is optimized for the quantitative analysis of the relationship between endothelial cell polarity and hemodynamics in the neonatal mouse retina. However, the software can be easily extended to study the relationship between blood flow and other cellular/molecular processes relevant to vascular morphogenesis, including but not restricted to gene expression patterns, changes in endothelial cell morphology, proliferation and apoptosis rates, changes in vessel caliber, or recruitment of mural cells. The only requirement is the ability to image and quantify the signal under investigation and compare it with the spatial distribution of flow parameters computed with the HemeLB CFD solver. This approach was developed for one of the most widely used animal models of angiogenesis: the mouse retina. However, other tissues of interest have more elaborate vascular architectures involving complex 3D vessel configurations. In the future, we plan to extend PolNet to include capabilities to segment and simulate hemodynamics in tissues and organs displaying a highly 3D vascular organization. With the advent of improved clearing techniques (19) and new imaging techniques (20), PolNet will be a powerful analysis method to address the complexity of endothelial cell biology at the network level in intact organs.

## Author Contributions

M.O.B., M.L.J., P.V.C., H.G., and C.A.F. designed the research; M.O.B., M.L.J., R.W.N., A.P., and C.A.F. performed the research; R.W.N. and A.P. contributed analytic tools; M.O.B., M.L.J., A.P., and C.A.F. analyzed the data; M.O.B., M.L.J., R.W.N., A.P., P.V.C., H.G., and C.A.F wrote the manuscript.

## Supporting Material

Document S1. Supporting Materials and Methods, Figs. S1–S4, and Tables S1–S3

Document S2. Article plus Supporting Material
