# Supplementary material for: PolNet: A Tool to Quantify Network-Level Cell Polarity and Blood Flow in Vascular Remodeling
Source: Biophys J. 2018 May 8;114(9):2052–8. doi: 10.1016/j.bpj.2018.03.032 (PMC5961748; doi:10.1016/j.bpj.2018.03.032)
Supplement: Document S1. Supporting Materials and Methods, Figs. S1–S4, and Tables S1–S3 [file mmc1.pdf]

Biophysical Journal, Volume *114*

Supplemental Information

PolNet: A Tool to Quantify Network-Level Cell Polarity and Blood Flow in Vascular Remodeling

Miguel O. Bernabeu, Martin L. Jones, Rupert W. Nash, Anna Pezzarossa, Peter V. Coveney, Holger Gerhardt, and Claudio A. Franco

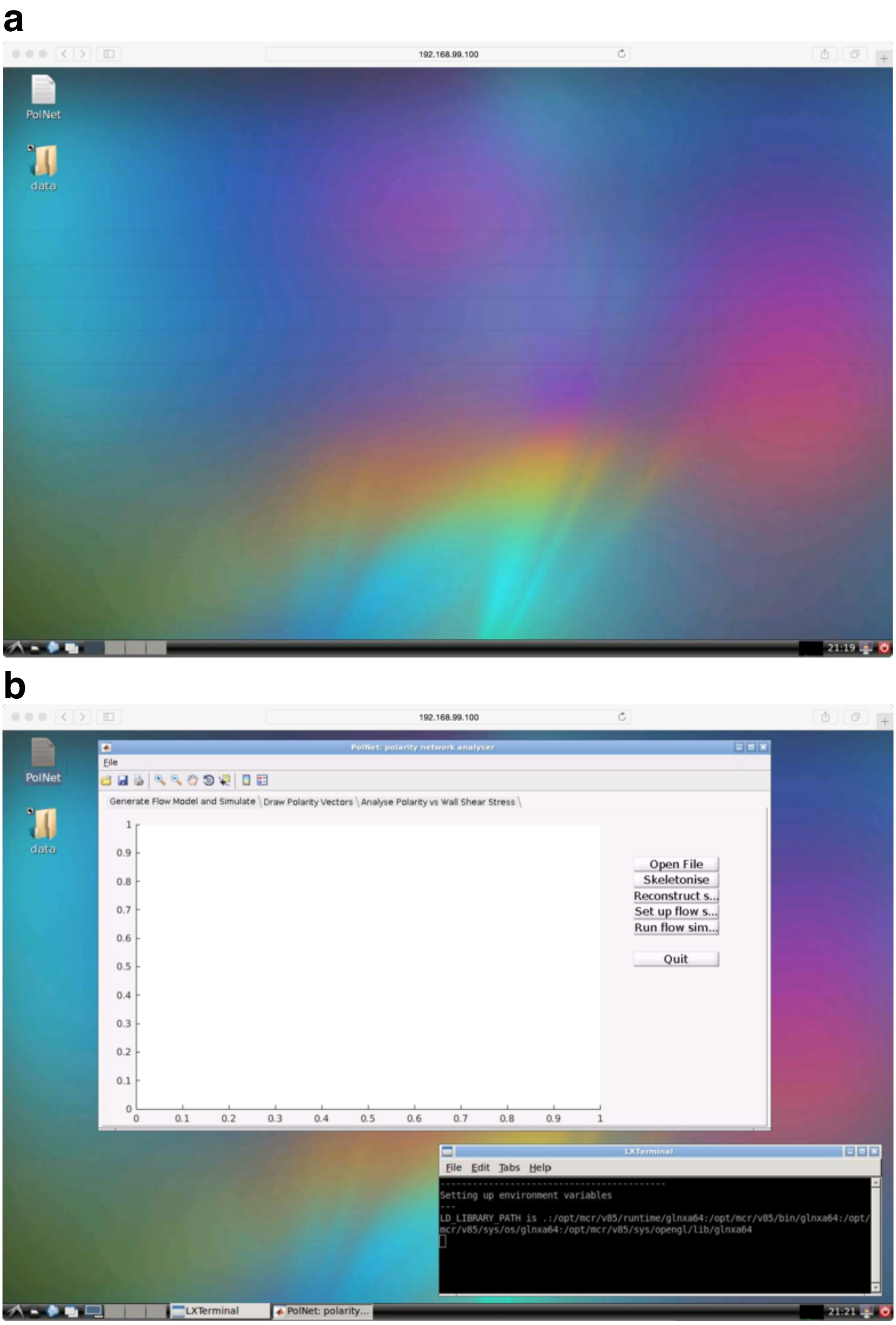

Figure S1: Docker and PolNet installation: a) Web browser displaying the Desktop of the PolNet container, which contains a launcher for the PolNet application and a link to the host data folder mounted on the container file system; b) PolNet application running inside the container, including the main graphical user interface (GUI) window and a console displaying a message log. The different components of the application are organised in three tabs.

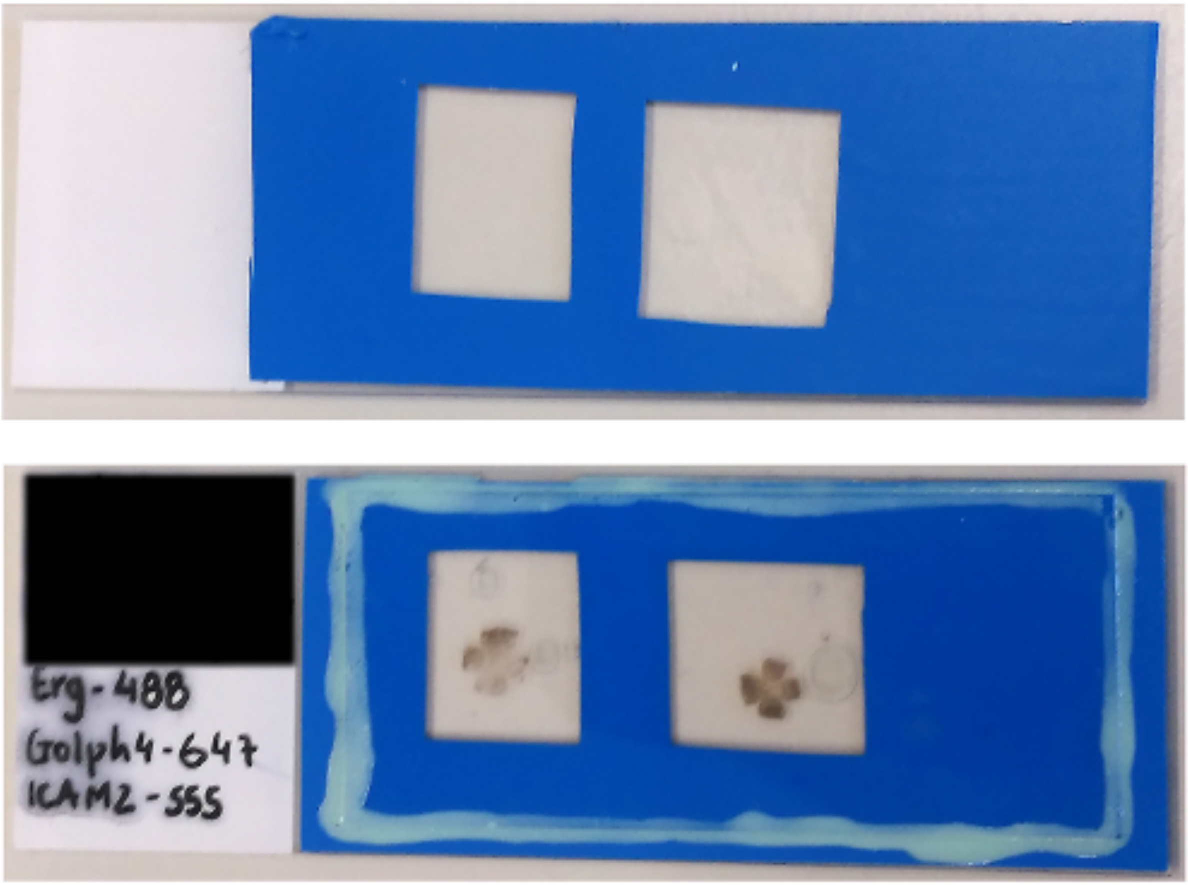

Figure S2: Mounting mouse retinas with minimal 3D squeezing: a) Glass slide pre-processed with microchambers with isolation tape centrally cut with forceps; b) Mounted retinas according to the protocol steps 9-15.

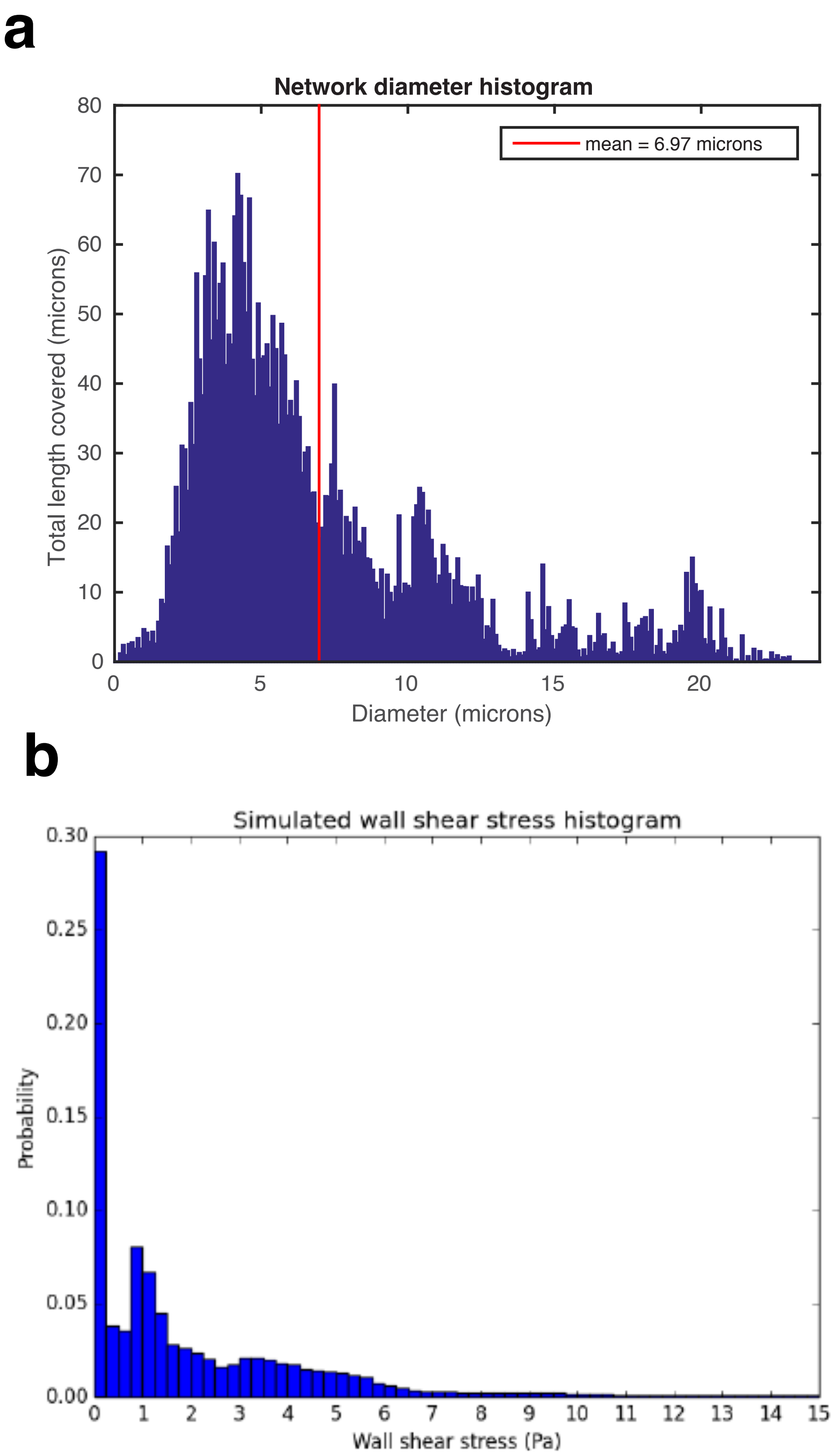

Figure S3: a) Vessel calibre histogram for the computed skeleton. b) Wall shear stress (WSS) histogram. The large proportion of WSS values in the 0-0.25 Pa bin correspond to blind-ended vessels due to recent vessel regression events or those in the region highlighted in Figure 1b.

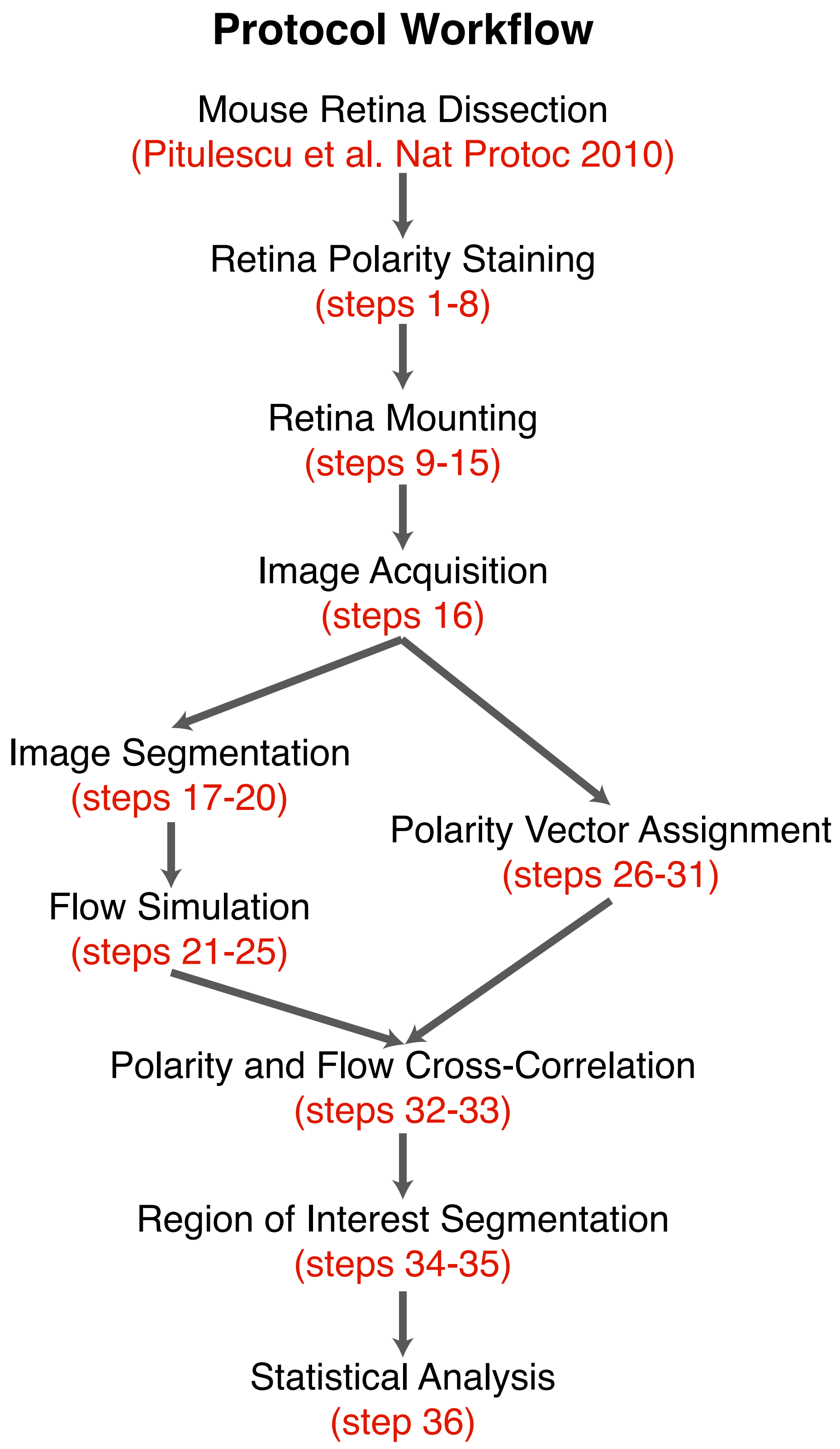

Figure S4: Graphical representation of the protocol workflow. Each of the protocol main stages is listed along with the steps comprising them (between parenthesis). An arrow between two stages indicates a data dependency between the stages at the tip and the base of the arrow.

Table S1: URL to Docker installation instructions for several OSs at the time of writing.

**Host's Operating System**	**Installation instructions**
Windows	https://docs.docker.com/docker-for-windows/install/
macOS	https://docs.docker.com/docker-for-mac/install/
Ubuntu	https://docs.docker.com/install/linux/docker-ce/ubuntu/

Table S2: Strings representing paths on the Docker terminal for several OSs. * Note: At the time of writing, Docker only supports mounting host directories within C:\Users (Windows) or /Users (OS X). Blank spaces in the directory names are discouraged.

**Operating System**	**<path_data> substitution example**
Windows*	//c/Users/polnet/polnet_data (corresponds to C:\Users\polnet\polnet_data)
OS X*	/Users/polnet/polnet_data
GNU/Linux	/home/polnet/polnet_data

Table S3: Quick reference of PolNet commands and URLs.

**Operation**	**Command/URL**	**Comment**
Launch PolNet container	docker run -i -t -p 6080:80 -v <path_data>:/data mobernabeu/polnet	Substitute <path_data> with the name of the directory containing the data that the user wants to analyse with PolNet. See Table S2 for formats.
PolNet container URL	http://127.0.0.1:6080	
Stop PolNet container	CTRL+C	Use in the terminal from which the container was launched.

SUPPLEMENTARY MATERIAL A: MATERIALS AND SETUP

Reagents

Rat anti-mouse ICAM2 (polyclonal antibody, detects luminal endothelial membrane domains; BD Biosciences, cat. no. 553326)
Mouse anti-rat GM130 (monoclonal antibody, detects Golgi apparatus; BD Biosciences, cat. no. 553326)
Rabbit anti-mouse ERG (polyclonal antibody, detects endothelial cell nuclei; Santa Cruz Biotechnology, cat. no. SC-353)
Alexa Fluor 647 (donkey anti-rat antibody; Invitrogen, cat. no. A21247)
Alexa Fluor 488 (donkey anti-rabbit antibody; Invitrogen, cat. no. A21208)
DAPI [4,6-Diamidino-2-phenylindole dihydrochloride; Sigma-Aldrich, cat. no. D9542]
Vectashield (antifade mounting medium; Vector Laboratories, cat. no. H-1000)
Insulation Tape (AT7 25mm x 33m PVC Electrical Insulation Tape; Advance Tapes, cat. no. 10003626)
Nail polish (any commercially available)
BSA (bovine serum albumin; Sigma-Aldrich, cat. no. A4378-25G)
Sodium Deoxycholate (Sigma-Aldrich, cat. no. D6750) CAUTION! It is harmful if inhaled and swallowed. It is irritating to eyes, respiratory system and skin.
PBS (Dulbecco's PBS, powdered; Biochrom AG, cat. no. L182-10)
FBS (Foetal Bovine Serum; GIBCO cat. no. 10500-064)
Sodium chloride (Carl ROTH, cat. no. 3957.1)
Sodium citrate tribasic dihydrate (Sigma-Aldrich, cat. no. C8532)
Triton X-100 (Sigma-Aldrich, cat. no. T8787) CAUTION! It is harmful if swallowed and can cause serious damage to the eyes.

Equipment

Reaction tubes (1.5, 2, 15, and 50mL)
Laser Scanning Confocal microscope (Zeiss LSM780, Zeiss)
Dissecting instruments: dissection forceps no. 5; short-blade scissors; spring scissors (Fine Science Tools, cat. nos. 91150-20; 14084-08; 15003-08)
Dissection microscope (Zeiss Stemi 305)
Microscope coverslips (Fisher Scientific, Menzel-Gläser, cat. no. 9161028)
Microscope glass slides (Fisher Scientific, Menzel-Gläser, cat. no. 9161145)
Glass Petri dishes
Thermomixer (Eppendorf Thermomixer C). Settings: cycles of 30sec at 400rpm followed by on hold for 5min.
Thermoblock (Eppendorf)
Bitplane Imaris Software / FIJI
Adobe Photoshop software (running on computer with a minimum of 2GB of RAM memory)
Docker software with PolNet container (see Supplementary Material Section B-Timing for suggested computer specifications)

Reagent setup

PBS solution: Dissolve 95.5 g of Dulbecco's PBS powder in 10 liters of ddH2O. Autoclave and store the aliquots at RT for 1 year.
Triton X-100 10% (vol/vol) solution: Dilute 1mL of Triton X-100 in 10mL of PBS 1X. Vortex vigorously until complete homogenisation. Store at 4 °C for 1 year.

Sodium Deoxycholate 5% solution: Dilute 0.5g of Sodium Deoxycholate in 10mL of PBS 1X. Vortex gently until complete homogenisation. Store at 4 °C for 1 year.
PBT solution: Mix 2ml of 10% (vol/vol) Triton X-100 and 98 ml of PBS 1X.
Claudio blocking buffer (CBB): Mix 0.3 g of BSA, 0.1mL FBS, 0.5ml of Triton X-100 10% (vol/vol) solution, 0.1mL of Sodium Deoxycholate 5% solution with 92 ml of PBS 1X. Prepare fresh and store at 4 °C for each experiment.
Claudio antibody buffer (CAB): Mix 25mL of CBB with 25mL of PBS 1X. Prepare fresh and store at 4 °C for each experiment.

Equipment setup

Installing and running PolNet

1 The first step in order to install and run the PolNet application is to install the Docker software in the user's machine, which we will refer to as the host machine in these instructions. Docker allows the execution of so-called containers that emulate a complete operating system and come with pre-installed applications.
Docker's installation process is well documented in the Docker website (https://www.docker.com, at the time of writing) and varies slightly depending on the host machine's OS. For this reason, we point the user to the specific installation instructions maintained by the Docker developers. See Table S1 for the URL corresponding to three of the most widely adopted OSs. Briefly, in a macOS environment, the user should download the installer from the Docker Stable channel (see link in URL provided in Table S1), double-click the downloaded Docker.dmg file to open a new window, and in this window drag the Docker icon to the Applications folder. The interested reader can refer to a detailed Docker tutorial at https://docs.docker.com/get-started/ but this is not strictly required by PolNet users not needing to make changes to the Docker container configuration.

2 In Windows and macOS, launch the Docker application. The first time this is done, the user may be asked to give Docker privileged access to the machine in order to install additional components. A Docker icon will appear in the status bar.

3 Open a terminal and type the following command to launch the PolNet container:
docker run -i -t -p 6080:80 -v <path_data>:/data mobernabeu/polnet
substituting <path_data> with the name of the directory containing the data that the user wants to analyse with PolNet. Table S2 shows some possibilities assuming that the user account name is polnet and that the user has created a directory called polnet_data in his or her home directory. This can be easily adapted to any other user account or directory names.

If this is the first time that the command is run, it will take some additional time to complete since the PolNet container needs to be downloaded.

4 Open a web browser and connect to the address **http://127.0.0.1:6080**. The web browser window will now display the desktop of the PolNet container (see Figure S1a).

5 Double click on the PolNet icon located in the container desktop. This will open two windows: one with the PolNet application Graphical User Interface (GUI) and one

with a console displaying messages associated with the application. The user will interact with the former, the latter functions as an execution log and can be used to diagnose errors in the GUI (see Figure S1b for an example).
Additionally, the user can double click on the data icon in the desktop to check that the directory containing the data to be analysed in the host machine has been correctly mounted in the container.

6 In order to stop the Docker container, the user can close the browser window and then go to the terminal where the container was launched and press **CTRL+C**. If the user wants to restart the container go to Equipment Setup Step 3.
IMPORTANT: The user must store all the important output files in the /data folder of the container (which maps to the host directory specified in Equipment Setup Step 3. Files stored in any other location within the container will be lost when the container is stopped.

SUPPLEMENTARY MATERIAL B: PROCEDURE AND TIMING

Procedure

Figure S4 presents a graphical representation of the protocol workflow with the main protocol stages along with the step numbers comprising them. Arrows between stages indicate data dependencies.

Labelling of the retinal vasculature

1 Collect and fix eyes according to the protocol described in (Pitulescu et al. 2010). However, we recommend a fixation protocol with 2% Paraformaldehyde for 5H at 4 °C in the thermomixer (see Supplementary Material Section A-Equipment for settings) or other means for gentle agitation/mixing. Proceed to dissection of retinas according to (Pitulescu et al. 2010) instructions.

2 Replace the PBS solution with 1mL of CBB to permeabilise/block retinas. Incubate the retinas 2 hours at room temperature in the thermomixer.
CRITICAL STEP CBB is a special buffer that enhances permeabilisation and specificity of antibody binding. This buffer is important to obtain high-contrast fluorescent images for processing.

3 Discard the CBB blocking solution and incubate primary antibodies (anti-Icam2 [dilution 1:200] and anti-Erg [dilution 1:100]) in CAB at 4 °C overnight in the thermomixer. Use 100uL per retina in a round-bottom 2ml eppendorf.

4 Next day, wash retinas 4X 30min with 2mL with PBT at room temperature in a thermomixer.

5 Incubate secondary antibodies (including antiGM130-Alexa555 [dilution 1:800], anti-rabbit Alexa488 [dilution 1:400], and anti-rat Alexa647 [dilution 1:400]) in CAB at 4 °C overnight in the thermomixer. Use 100uL per retina in a round-bottom 2ml eppendorf.

6 Next day, wash retinas 4X 30min with 2mL with PBT at room temperature in a thermomixer.

7 (OPTIONAL) After the final wash, incubate 30min with DAPI 1X solution treatment at this stage, if nuclear staining is used. DAPI staining can help less experienced researchers to distinguish endothelial from non-endothelial Golgi complexes.

8 Wash the retinas once 15min at room temperature in a thermomixer. Proceed to the mounting step.

Mounting mouse retinas

9 Mount the retinas on glass slide, pre-processed with microchambers, as showed in Figure S2a. CRITICAL STEP Microchambers allow enough spacing between the glass slide and the coverslip to allow optimal geometry of the retina. This enables that retinas remain flat-mounted but not squeezed. Squeezing causes vessels to collapse or to distort, which can be problematic for segmentation and flow simulation.

10 Label the glass microscope slides. Transfer both retinas of the same pup to the glass slide using a transfer pipette.

11 Use forceps to position retinas with the inner side containing the vessels facing up (as exemplified in Pitulescu et al. 2010). Carefully remove any debris sticking to the samples. Remove excess PBS solution around the retinas using a 200-µl pipette.

12 Flatten the samples by gently pressing the outer edge of the retinas with the forceps. If necessary, remove more PBS solution from below the edge of the retina. IMPORTANT Retinas must never dry out completely during this procedure.

13 Pipette 60 µl of Vectashield onto a microscope coverslip. Invert the coverslip and place it over the retina. Compress gently to flatten the retinas and spread the mounting media.

14 Absorb any excess of liquid on the edges of the coverslip with a tissue. Seal the edges all around the coverslip with nail polish, as shown in Figure S2b.

15 Keep the slides at room temperature for 1–2 h. They are now ready for imaging or for storage at 4 °C. The best image quality can be obtained within the first 1 week. Retinas, if appropriately mounted, can be stored for up to 6 months.

Retinal vascular network image acquisition and processing

16 Retinas are imaged using a confocal microscope (e.g. Zeiss Laser Scanning Confocal microscope LSM780 with motorized stage or a Zeiss Cell Observer spinning disk microscope). Entire quadrants of the mouse retina are imaged using the tile-scan imaging mode with an oil-immersion 40X objective lens (NA 1.30 at 1,024 × 1,024 pixels). Optimal Nyquist sampling Z-sections are determined directly by the microscope software (e.g. Zeiss ZEN software). Choose regions with no visible damage in the vascular network, and comprising at least the following configuration: artery-vein-artery or vein-artery-vein (this is critical for modelling flow as it provides the inlet and outlet). 3 different channels should be acquired separately: 1) ICAM2 staining; 2) Erg staining; 3) GOLPH4 staining. An additional 4th channel, corresponding to the DAPI staining is also included in case users decided to proceed with Step 7. We would like to highlight that the image that we have included in our protocol (Figure 1a) is just a subset of an entire network. We used this reduced minimalist view to be easier to illustrate the method to readers, although it is not a vessel configuration that should be used in actual experiments. Users should use entire retina plexuses with artery-vein-artery or vein-artery-vein configurations, as shown in Franco et al 2015; Franco et al 2016.

17 Acquired images are processed using FIJI software.

i. Open image using Bio-formats importer: Pluggins>Bio-formats>Bio-formats importer

ii. Create Z-projection: Image>Stacks>Z-project ('Projection type': maximum intensity)

iii. Make Composite image: Image>Color>Make Composite

18 The FIJI composite image is then saved as a .tiff (TIFF1).

i. Save image as RGB: Image>Type>RGB; then File>Save

This image will be used to draw axial polarity maps (steps 26-31) of endothelial cells using the PolNet software (Figure 2).

19 In parallel, the layer corresponding to the ICAM2 immunostaining is further processed to create a binary mask to be used to reconstruct the 3D vascular network for the flow simulations (see Figure 1). The workflow involves the following steps:

i. On Composite Image, perform separate Channels: Image>Color>Split Channels

ii. Select ICAM2 channel and discard the other channels.

iii. Threshold ICAM2 signal (Image>Adjust>Threshold): Adjust the threshold so that all the vasculature appears red. Non-vascular dot-like noise is likely to be selected around the image but will be then removed in the next steps. After, selecting the correct threshold, click apply. This will generate a B&W image, where vessels are in black. Make sure that the option "Dark background" in the threshold window in not selected.

iv. To fill small gaps and smoothen the network segmentation and remove noise perform the following three operations:

a. Process>Noise>Remove Outliers; with the following settings: 'Radius': 5.0px; 'Threshold': 50; 'Which Outliers': Dark; then click OK;

b. Process>Binary>Dilate;

c. Process>Binary>Erode.

v. Remove unconnected objects with Analyze>Analyze Particles and the following settings: 'Size': 250-Infinity; 'Circularity': 0.00-1.00; 'Show': Masks; untick all optional boxes. This generates a B&W image of the vascular plexus.

vi. Save the layer as .tiff (TIFF2), taking care of not saving the other layers on the same file.

20 At this step, users should have 2 separate .tiff files, one (TIFF1) containing 3 layers for the specific stainings (Golph4; Erg; and ICAM2); and a second one (TIFF2) containing the segmentation of the ICAM2 staining. These two files should be then copied into the host data directory used by Docker (see Supplementary Material Section A-Equipment Setup for details) to be further processed with PolNet.

Flow model creation and simulation

The following steps are performed in the PolNet software. See Supplementary Material Section A-Equipment Setup for installation and execution instructions. All the functionality in this subsection can be found on the first tab "Generate Flow Model and Simulate".

21 Open File will present a file selection dialog. Select the black and white mask generated in Step 20 and accept. PolNet will now display the chosen image.

22 Skeletonise will first open a dialog box asking the user to provide the pixel/micrometre ratio of the image. This can be obtained from the imaging setup described in Step 16. Once provided, PolNet will compute the skeleton of the image (and the associated radii of each vessel segment) and overlay it on top of the original image (the original image may be color inverted if it was provided with the opposite

lumen/background tissue color convention) (Figure 1c). The user should inspect the image (use the zoom in/out and pan buttons on the main icon bar) and check that a single skeleton line appears on each vessel segment. If spurious skeleton branches have appeared or the inlet/outlet segments have been shortened in excess go to the Troubleshooting section for instructions on how to tune the configuration of the skeletonisation algorithm.

23 Reconstruct Surface will reconstruct a three-dimensional (3D) model of the vessels luminal surface based on the skeleton and radii computed in the previous step. It will also display a network radii histogram (see Figure S3a) for the user to validate the computed radii against manual measurements obtained directly from the imaged plexus. This step can be time consuming. Because no additional visual output is generated, a message is displayed informing that the operation is running. The user should not try to interact with the application until this message disappears.

24 Setup Flow Simulation will open a new window with the HemeLB Setup Tool and will transfer control to it. In this step, the user will graphically configure the location of the inlets and outlets in the network, provide information about the boundary conditions of the simulation, and generate the discretisation of the luminal space required to simulate flow with HemeLB (Figure 1d). The inlets and outlets are the vessel segments that connect the plexus under study to the rest of the cardiovascular system and are responsible for feeding and draining blood, respectively. The GUI is split into two main parts. The right-hand-side panel is a rendering window that allows the user to zoom, pan, and rotate the 3D model generated in the previous step. The left-hand-side panel presents the main controls used to interact with the application. Most of the information in the left-hand-panel has been prepopulated for the user. The user must specify at least one inlet and one outlet for a simulation to be meaningful as follows:

i. Click on Add Inlet. This will create a new entry in the Inlets and Outlets text box.

ii. Select this new entry and provide an approximate radius for the inlet. This is the radius of a circle inscribed in the square to be displayed. The value can be updated at any time.

iii. Click the Place button and using the mouse to select the location of the inlet region on the 3D model on the right-hand-side window. This is only an initial location that will be fine-tuned in the next step. The user can click on the vessel surface to change the initial location as many times as required. Once the user is satisfied with the location click on the Finish button that will have replaced Place.

iv. Using the right-hand-side window the user can fine tune the location and orientation of the square (note the normal arrow going through the plane. Some of the options are:

a. Drag and drop the plane to move it around. This can be combined with zooming, panning and rotation of the 3D rendered scene to achieve precise positioning.

b. Drag and drop the normal to change the orientation of the plane. The normal vector has arrowheads at both of its ends. The user must ensure that the vector points into the geometry (i.e. an inward facing normal).

c. Drag and drop any of the corners of the square to change the square size.

v. Edit the Pressure text boxes to define the mean pressure at the inlet (first box) and optionally a sinusoidal pressure wave of a given amplitude (second box), phase (third box), and 1 Hz frequency to be added to the mean pressure. In (Bernabeu et al. 2014; Franco et al. 2015), we used a constant inlet pressure of 55 mmHg. See Section Limitations for a discussion on the uncertainty of this choice. The pressure at the outlet will be typically set to 0 mmHg, so that the inlet pressure is equivalent to the total pressure drop across the domain.
Click on the Place button next to Seed position and select a point along the luminal surface contained within any pair of inlet/outlets. A blue sphere will appear, if successful. Click on the Finish button that has appeared where the original Place button was.
Once the previous steps have been carried out, the user can press the Generate button to generate the set of files necessary to run a HemeLB simulation based on the information captured by the GUI. A pop up window will be displayed indicating that the operation is still running. Once the flow model has been generated the user can close the Setup Tool window. The program will then return control to the main PolNet window.

25 Run Flow Simulation will run the flow simulation configured in the previous step. A pop up window will ask the user for the number of CPU cores to be used by the parallel CFD solver. A suggestion is provided based on the number of CPU cores available on the hardware running PolNet (please refer to the Troubleshooting section, if the suggested figure is smaller than the number of physical CPU cores available). Because no additional visual output is generated, a message is displayed informing that the operation is running. After the simulation has completed a pop up window will inform the user whether the simulation has completed successfully or not. See the Troubleshooting section for the most common causes for a simulation to fail. If the simulation terminated successfully, a WSS histogram (see Figure S3b) will be presented to the user to visually validate the results obtained (please see Section Results for some experimentally measured reference values). Note that the flow results are stored in the “results/” subdirectory newly created within the directory containing the original luminal mask used. If a directory with that name already exists, the user will be asked, as a safety measure, whether PolNet should overwrite it or not.

Drawing polarity vectors

The following steps are performed on the PolNet software. See Supplementary Material Section A-Equipment setup for installation and execution instructions. All the functionality in this subsection can be found on the second tab “Draw polarity vectors”.

26 Open File will present a file selection dialog. Select the microscope image showing the nuclei/Golgi markers generated in Step 19 and accept. PolNet will display the image.

27 (Optional) Load Position Data will present a file selection dialog. Select a .csv file with a set of polarity vectors previously saved. This will allow the user to resume a polarity delineation session previously saved.

28 Start Selecting Cells starts a polarity delineation session (or resumes it, if Load Position Data was performed). The mouse pointer will change to a crosshair which allows the user to place, on top of the image being displayed, consecutive pairs of points corresponding to the approximate centre of mass of the nucleus and Golgi of any given cell. Every time a new pair of points is added, an arrow connecting them will be automatically drawn. This arrow defines the polarity vector of a given cell (see Figure 2a and b). The user can press the ESC key at any time to quit the delineation session, which will change the mouse pointer back to its original form. The user can now interact with the application (for instance zooming in/out, panning or using Delete Last Point) and then press Start Selecting Cells in order to resume the delineation session. The final image (Figure 2c) with the positional information of all vectors can be save using the save option of MATLAB.

29 (Optional) The Crosshair Color drop down menu allows the user to change the color of the crosshair in order to enhance contrast against the background image.

30 Save Position Data will present a file selection dialog to save a .csv file with the polarity vectors drawn. This file can be used to: a) resume the delineation session at any time with Load Position Data or b) compare polarity against flow as described in the following section.

31 Delete Last Arrow allows the user to remove the last polarity vector drawn. This operation can be performed multiple times to correct delineation errors.

Polarity vs wall shear stress analysis

The following steps are performed on the PolNet software. See Supplementary Material Section A-Equipment Setup for installation and execution instructions. All the functionality in this subsection can be found on the third tab “Analyse Polarity vs Wall Shear Stress”.

32 Get flow info at cell nuclei will first present a file selection dialog. Select a .csv file with a set of polarity vectors saved in Step 31. PolNet will calculate the value of the flow variable selected in the dropdown menu below (one of wall shear stress, velocity or pressure) at each cell nucleus given in the file. If the user has selected Use last simulation results, PolNet will use the latest simulation results generated in Step 26. Otherwise it will let the user choose a directory containing an existing results subdirectory to calculate the flow at the cell nuclei. This feature is useful if the user needs to analyse multiple delineations (or corrections to an existing one) without having to rerun the flow simulation multiple times.

33 Display mask and vectors will present a file selection dialog for the plexus mask image (.tif extension), immediately followed by a file selection dialog for the file containing the nucleus-Golgi positions (.csv extension). The corresponding flow file is automatically identified and loaded and the vectors corresponding to the nucleus-Golgi positions (blue) and flow magnitude and direction (red) are overlaid onto the plexus mask (Figure 3a). Two plots are generated to give an overview of the dataset: a histogram showing the distribution of scalar product values for all cells and a polar histogram showing the relative angles between the cell and flow vectors (Figure 3b). The distribution of angles is tested for circular uniformity using the Omnibus test

(Berens 2009) under the null hypothesis that the population of angles are uniformly distributed around the circle (i.e. the angles are randomly distributed). The result of the test is displayed in the same figure.

34 Subdivide into regions asks the user for a number of regions to subdivide the image into. The user then draws a closed polygon for each region by left-clicking to set a series of vertices and then either left-clicking on the first point to close the polygon or right-clicking to join the most recent vertex to the first to close the polygon. The cells contained within each polygon are assigned the corresponding label. If regions overlap, the cell belongs to the first region to which it was assigned. The regions can be adjusted by dragging the vertices and extra vertices can be added by holding the “a” key while clicking on an edge. In the case of overlapping regions, the most recently drawn region takes precedence when selecting the vertices/edges. Clicking on the Subdivide into regions button again will restart the process, allowing the user to choose a different number of regions.

35 Analyse Regions will perform the subdivision, colouring the regions according to their grouping whilst preserving the precedence rules for overlapping regions previously described (Figure 3c). A scatter plot is generated showing the flow parameter plotted on the x-axis against the scalar product on the y-axis with the points color-coded according to their region. A series of polar histograms are generated showing the angular distribution between the cell and flow vectors for each region (Figure 3e). If the region polygons are adjusted, clicking Analyse Regions again will redraw the regions to indicate the new groupings and re-generate the series of plots.

36 Save Data opens a file dialog for the user to choose a directory location and filename to store the generated data as a .csv file. This allows the user to perform additional analyses currently not implemented in PolNet App, but important for statistical analysis of samples, including the shear stress sensor analysis (Figure 3d), and scalar product histogram analysis (Figure 3f and g). The output file has a row for each cell and 7 columns, corresponding to: Nucleus x-position; Nucleus y-position; Cell vector length; Flow vector length; Angle between vectors; Scalar Product of vectors; Group. Note that cells that were not including within any of the selected regions are recorded with a Group value of zero. Group are numbered 1 to max number of regions according to the order in which they were selected.

Timing

In this section, we list the most time-consuming tasks in the protocol along with an estimate of the time required to complete them. The tasks not listed here are considered trivial. Many of the computational tasks have a variable duration, which depends on the size and complexity of the vascular plexus being analysed. In those cases, task duration is given in time per unit area of tissue being analysed. To estimate this value we benchmarked PolNet with the example dataset provided with this paper (total area 0.1416 mm2, see Section Results). We benchmarked the code on a MacBook Pro laptop (Apple Inc.) with a 2.5 GHz Intel Core i7 processor, 16 GB of 1600 MHz DDR3 RAM, and a NVIDIA GeForce GT 750M graphics card with 2048 MB of memory. The code was run on a PolNet developer install rather than through a Docker container (see Section Availability and Future Directions for instructions on how to perform a developer install).

Installation

- Download and install Docker: 2-5 minutes depending on internet connection speed.
- Download PolNet container: 3-15 minutes depending on internet connection speed.

Sample preparation, imaging, and postprocessing:

- Labelling of the retinal vasculature: 3 days
- Mounting mouse retinas: 10 minutes per retina
- Retinal vascular network image acquisition and treatment: 4 hours

Flow model creation and simulation:

- Skeletonise: 40 seconds/mm2.
- Reconstruct surface: 13 minutes/mm2.
- Setup flow simulation: 32 minutes/mm2.
- Run flow simulation: 84 hours/mm2 with 4 CPU cores. Additional cores will reduce execution time for as long as a minimum number of flow domain discretisation points per core is ensured. The interested reader can refer to (Groen et al. 2013) for more details about the performance of the flow solver.

Drawing polarity vectors:

- Select cells: 10-15 seconds per endothelial cell nucleus-Golgi pair.

Polarity vs wall shear stress analysis

- Get flow info at cell nuclei: 10 seconds/mm2.
- Analyse Regions: 30 seconds/mm2.

SUPPLEMENTARY MATERIAL C: EXAMPLE DATASET

One example Golph4-Erg-ICAM2 colour image (red-green-blue, respectively).

One example ICAM2 binary image.

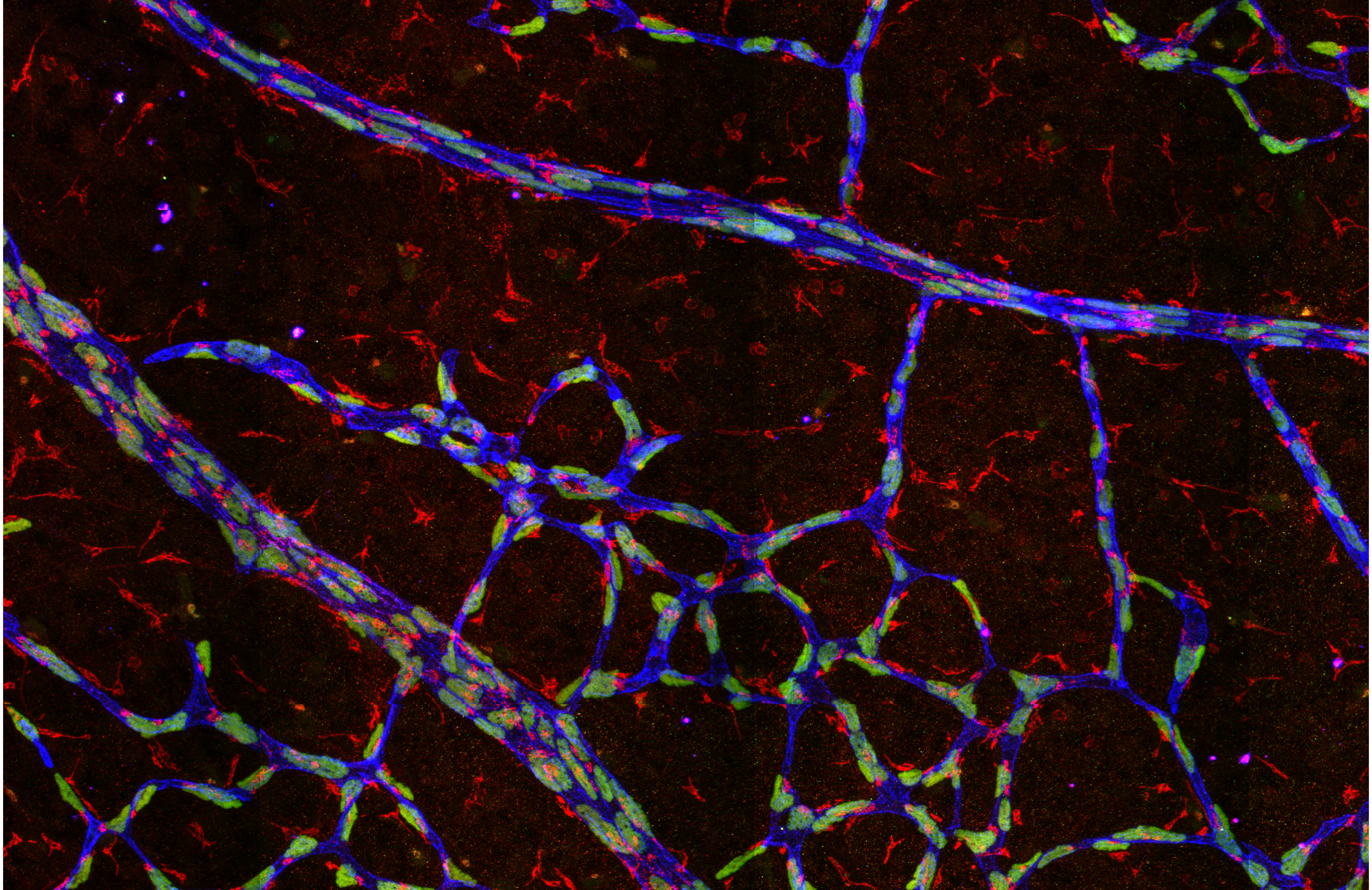

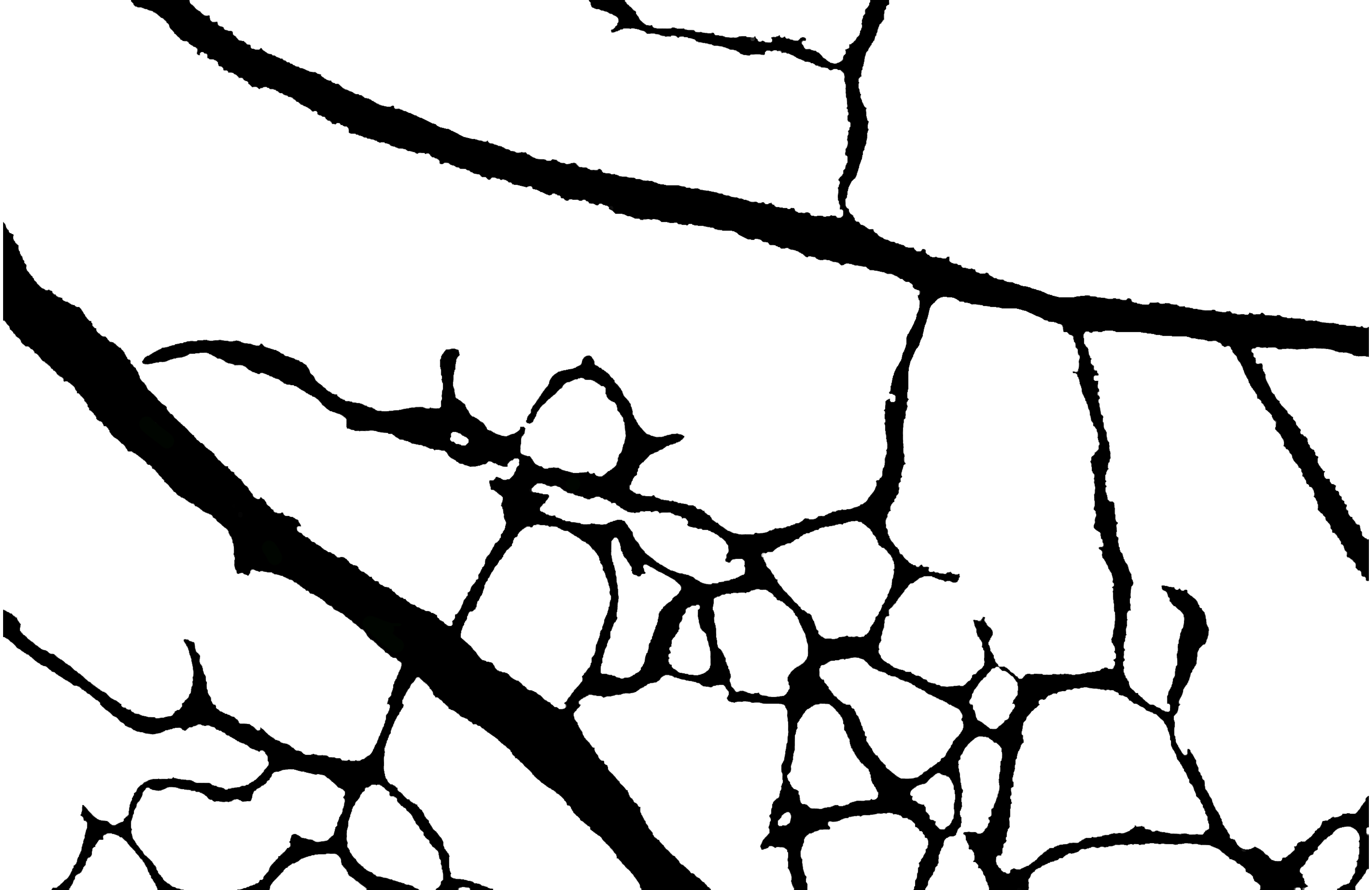

SUPPLEMENTARY MATERIAL D: TROUBLESHOOTING

Problem	Solution	Details
Immunostainings are of poor quality	Verify that all reagents are fresh. Use fresh tissue samples. Refer to (Pitulescu et al. 2010) for additional troubleshooting.	One or more of the immunostainings did not work as expected. Possible issues include, but are not restricted to: no staining; staining with low intensity; cross-reaction between primary or secondary antibodies; non-homogenous staining in the sample.
Plexus reconstruction has missing or disconnected vessels.	Increase the x-, y-, and z-resolution parameters in the configuration window of “Reconstruct surface”. Note than unnecessarily high values will lead to extend execution times for this step.	Depending on the image size and resolution, the “Reconstruct surface” operation may produce a luminal surface model with missing, or artificially narrow, vessels. This can be observed in the “Set up flow simulation” step or by visualising the intermediate .stl file produced during the “Reconstruct surface” step with an external visualiser (e.g. Paraview). This problem arises when the resolution used to sample the network luminal space defined by the image skeleton and radii is not sufficient to resolve all the fine detail in the network.
HemeLB simulation fails due to numerical instability in the flow solver	This issue could indicate an incorrect configuration of the inlets and outlets (e.g. pressure drop is too high). If this is not the case, the user can increase the resolution of the flow domain discretisation by increasing the parameter “Minimum diameter in lattice units” in the configuration window of the “Reconstruct surface” operation.	This problem can be diagnosed if a “Flow simulation failed” message appears after the “Run flow simulation” operation and the PolNet terminal displays the error message “Unstable simulation. Aborting”. This problem is often caused by a choice of flow domain discretisation parameters, which are not sufficiently fine to be able to handle accurately the flow velocities in the domain.
“Reconstruct surface”, “Set up flow simulation” or “Run flow simulation” fail and the PolNet terminal shows a “std::bad_alloc” message.	Configure Docker to have access to a larger amount of memory in the host environment (Docker Preferences, Advanced, Memory) or use a different computer with more available memory.	This problem suggests that PolNet does not have access to enough memory to run one of these stages. This problem arises when the flow model is too big to fit in memory.
