# Supplementary material for: PolNet: A Tool to Quantify Network-Level Cell Polarity and Blood Flow in Vascular Remodeling
Source: Biophys J. 2018 May 8;114(9):2052–8. doi: 10.1016/j.bpj.2018.03.032 (PMC5961748; doi:10.1016/j.bpj.2018.03.032)
Supplement: Document S2. Article plus Supporting Material [file mmc2.pdf]

Biophysical Journal
Computational Tool

Biophysical Society

PolNet: A Tool to Quantify Network-Level Cell Polarity and Blood Flow in Vascular Remodeling

Miguel O. Bernabeu,[1,2,*] Martin L. Jones,[3] Rupert W. Nash,[4,2] Anna Pezzarossa,[5] Peter V. Coveney,[2] Holger Gerhardt,[6,7,8,9,10] and Claudio A. Franco[5,*]

[1]Centre for Medical Informatics, Usher Institute, The University of Edinburgh, Edinburgh, United Kingdom; [2]Centre for Computational Science, Department of Chemistry, University College London, London, United Kingdom; [3]Electron Microscopy Science Technology Platform, The Francis Crick Institute, London, United Kingdom; [4]EPCC, School of Physics and Astronomy, The University of Edinburgh, Edinburgh, United Kingdom; [5]Instituto de Medicina Molecular, Faculdade de Medicina, Universidade de Lisboa, Lisboa, Portugal; [6]Max Delbrück Center for Molecular Medicine in the Helmholtz Association, Berlin, Germany; [7]Vascular Patterning Laboratory, Center for Cancer Biology, VIB, Leuven, Belgium; [8]Vascular Patterning Laboratory, Department of Oncology, KU Leuven, Leuven, Belgium; [9]German Center for Cardiovascular Research, Berlin, Germany; and [10]Berlin Institute of Health, Berlin, Germany

ABSTRACT In this article, we present PolNet, an open-source software tool for the study of blood flow and cell-level biological activity during vessel morphogenesis. We provide an image acquisition, segmentation, and analysis protocol to quantify endothelial cell polarity in entire in vivo vascular networks. In combination, we use computational fluid dynamics to characterize the hemodynamics of the vascular networks under study. The tool enables, to our knowledge for the first time, a network-level analysis of polarity and flow for individual endothelial cells. To date, PolNet has proven invaluable for the study of endothelial cell polarization and migration during vascular patterning, as demonstrated by two recent publications. Additionally, the tool can be easily extended to correlate blood flow with other experimental observations at the cellular/molecular level. We release the source code of our tool under the Lesser General Public License.

INTRODUCTION

The establishment of a functional, patterned vascular network is crucial for development, tissue growth, and physiology. Conversely, mispatterning of vascular networks contributes to the pathogenesis of several diseases, including arteriovenous malformations, hemangioma, hereditary hemorrhagic telangiectasias, or venous cavernomas. Sprouting angiogenesis is one of the mechanisms responsible for expanding vascular networks into avascular areas. This process only generates a dense and immature network of vessels that requires subsequent extensive remodeling to form a functional, hierarchically branched network—a process termed vascular remodeling (1). In contrast to sprouting, the molecular mechanisms regulating vascular remodeling are poorly understood. The forces exerted by blood on the luminal surface of the endothelium (most notably wall shear stress (WSS)) have been recognized as primary drivers regulating vascular remodeling (2–4). Recent work by the authors and others identified that endothelial WSS regulates endothelial cell polarity and cell migration, orchestrating vascular remodeling (5–8) and controlling vessel diameter (9–12). Thus, there is increasing interest in understanding mechanistically how hemodynamic forces impact endothelial response at the cellular and molecular levels.

The mouse retina is one of the most popular models to use when investigating the molecular mechanisms governing angiogenesis. However, the way in which blood flow influences the development of the retinal vasculature remains elusive. Progress is hampered by the limitations of current assays used to probe the relationship between hemodynamics and molecular response and the complexity of in vivo vascular connectivity. To date, researchers have primarily used in vitro microfluidic assays to study the impact of blood flow on endothelial cell biology, extrapolating these observations to explain phenotypic changes in mouse mutants. Even though some authors have been able to measure microvascular WSS in vivo (see Lipowsky et al. (13) for a survey), this has never been achieved, to the best of our knowledge, in the context of vascular morphogenesis in the mouse retina model.

Submitted December 18, 2017, and accepted for publication March 14, 2018.

*Correspondence: miguel.bernabeu@ed.ac.uk or cfranco@medicina.ulisboa.pt

Miguel O. Bernabeu and Martin L. Jones contributed equally to this work.

Editor: Eric Sobie.

https://doi.org/10.1016/j.bpj.2018.03.032

© 2018 Biophysical Society.

This is an open access article under the CC BY license (http://creativecommons.org/licenses/by/4.0/).

Our recent work on vascular remodeling established a strong connection between blood flow and endothelial cell polarity. We developed PolNet to be able to quantify the relationship between endothelial cell polarity and WSS. PolNet's image-processing algorithms and computational fluid dynamics (CFD) simulator were described and validated by Bernabeu et al (14). PolNet was then successfully used in two recent publications: 1) in Franco et al. (5), we showed that flow-induced cell polarization directs migration of endothelial cells away from low-flow segments, and 2) in Franco et al. (6), we showed that noncanonical Wnt signaling modulates the endothelial shear stress flow sensor during vascular remodeling. In this article, we focus on providing a step-by-step description of our experimental and computational protocols to facilitate the adoption of our polarity-WSS analysis. Furthermore, we provide a Docker-based installation of our tool and release its source code under the open-source Lesser General Public License.

Methods

The design and implementation of PolNet is better understood in the context of the complete protocol, comprising experimental and computational parts, used in Franco et al (5,6). The materials and setup are described in Supporting Materials and Methods, Section A, and a step-by-step protocol is provided in Supporting Materials and Methods, Section B. Briefly, samples of murine retinal plexus are collected at different postnatal (P) days, fixed, and labeled for the luminal surface (ICAM2), cell nuclei (Erg), and Golgi apparatus (Golph4). The retinal vascular networks are imaged and postprocessed to generate a binary mask from the ICAM2 channel and a second image with at least the Erg and Golph4 channels. These two images define the input to PolNet. An example data set is provided in Supporting Materials and Methods, Section C. Based on this, PolNet can be used to quantify the relationship between endothelial cell polarity (defined for each cell as the vector $\mathbf{p}$ originating from the center of mass of the nucleus and directed to the center of mass of the Golgi complex) and the direction and magnitude of the computed traction vector $\mathbf{t}$ (i.e., the product of the deviatoric stress tensor and the surface normal), which we will refer to as the WSS vector, for convenience. PolNet provides a graphical user interface for the user to perform the following three tasks: 1) to construct a flow model from the ICAM2 mask and use the HemeLB flow solver (15) to estimate the WSS across the whole network (as well as blood velocity, shear rate, and pressure), 2) to interactively delineate the cell polarity vectors for each endothelial cell in the plexus based on the Erg-Golph4 image, and 3) to statistically analyze the relationship between the cell polarity and WSS vectors, $\mathbf{p}$ and $\mathbf{t}$, respectively. PolNet currently offers the following analyses:

1) A directionality table shows the number and ratio of endothelial cells polarized against the flow direction.
2) A polar histogram shows the distribution of endothelial cell polarization angles in relation to the blood flow direction. Each plexus can also be subdivided into regions (e.g., artery, vein, capillary, and sprouting front), and each cell will be assigned to one of these vascular beds for further analysis. Multiple angular distributions can be subsequently compared using the Kuiper test to evaluate the likelihood that the two samples are drawn from the same underlying angular distribution.
3) The WSS sensor analysis allows us to bin the angular data according to the WSS magnitude to plot the proportion of cells oriented against the flow, which we define as having an angle of $180^\circ \pm 45^\circ$ with regard to the flow direction. We consider the threshold for polarization as the WSS value, leading to 60% of the endothelial cells being aligned against the flow direction.
4) Scalar product slope: we calculate the scalar product of the polarity and WSS vectors given by $\|\mathbf{p}\| \, \|\mathbf{t}\| \cos(\theta)$, where θ is the angle between them, which combines information about the length and relative angles of the vectors. By plotting the scalar product against $\|\mathbf{t}\|$, we can study and compare the plot slope, i.e., $\|\mathbf{p}\| \cos(\theta)$, between groups. A larger negative slope corresponds to a larger polarization effect for a given WSS, which is a surrogate measure for the sensitivity of cells to flow.

PolNet software repositories and contributions

Users interested in the software package in its current state (with just the functionality described in this article) are advised to follow the installation instructions in Supporting Materials and Methods, Section A, on how to install and run the current version of PolNet through the Docker platform. For advanced users or tool developers who wish to extend/adapt the software, PolNet can be easily modified to perform similar analyses involving the comparison of in silico flow estimates and other experimental observations at the cellular/molecular level. Table 1 summarizes the software repositories hosting the different components of PolNet.

Users interested in expanding the functionality of PolNet should obtain the code in the PolNet main repository and

TABLE 1 Software Repositories Hosting the Code Used to Build the PolNet Tool

PolNet Component	Description	Software Repository
PolNet main	graphical user interface and main pre- and postprocessing algorithms	https://github.com/mobernabeu/polnet
PolNet Dockerfile	Docker-specific instructions to create the PolNet container	https://github.com/mobernabeu/docker-polnet
HemeLB	flow model setup tool and CFD solver	https://github.com/UCL/hemelb

follow the installation instructions for developers. This will provide access to the source MATLAB (The MathWorks, Natick, MA) and Python scripts defining the graphical user interface and the main pre- and postprocessing algorithms. Users can then adapt the code to meet the requirements of their analyses and potentially contribute the changes back to the main repository. The user can build his/her own Docker container based on the instructions provided in our PolNet Dockerfile repository. Note that to run the HemeLB setup tool or solver as part of the PolNet developer version, a HemeLB developer installation is required. Please refer to the HemeLB repository for installation instructions. Similarly, users can report bugs and suggest new features to the PolNet developers (and other interested users) by creating new issues on the relevant GitHub repository.

Limitations of the method

Capillary network requirements. We employ the maximal intensity z-projection of a confocal microscopy image stack to segment the luminal space defining the flow domain. This implies that all the information in the z axis is projected onto a plane, and therefore, the method is only applicable to capillary beds where all the vessels can be considered coplanar in this projection. In the case of the neonatal mouse retina, this condition is fulfilled before P7, where a single nonoverlapping plexus covers the outer layers of the retina. If this condition is not fulfilled, vessels overlapping or going past each other at different depths will appear connected in the plexus segmentation, which will lead to inaccuracies in the simulated hemodynamics. This situation can be easily diagnosed if the image segmentation displays branching points with four afferent/efferent segments. Thus, this protocol is not suitable for mouse retinas beyond P7 or other vascular plexuses with complex three-dimensional (3D) organization. This would require segmentation of 3D image stacks, which is an aspect not yet implemented in the current version of PolNet. In addition, the method requires well-defined inlets (arteries) and outlets (veins) in the network for correct flow modeling. In the mouse retina, arteries and veins only become apparent after P3; thus, earlier stages cannot be reliably analyzed with PolNet. Special care also needs to be taken when analyzing mouse mutants strongly affecting vessel architecture. We advise against using PolNet in phenotypes such as 1) compromised arteries and/or venous differentiation, which lack clearly defined inlets/outlets; 2) dramatically affected vascular networks, including extremely overgrown capillary networks (e.g., Notch loss-of-function phenotypes (16)); and 3) artery-vein crossing phenotypes (e.g., Neuropillin(cyto)(Δ) retina (17)).

Polarity delineation. Assigning each Golgi apparatus to the corresponding endothelial nucleus is a manual operation and thus subject to human error. For accurate delineation, we recommend a computer setup with 1 or 2 monitors simultaneously displaying 1) the PolNet application for the nucleus and Golgi apparatus assignments and 2) an image analysis software (e.g., FIJI) to visualize each z-section of the z-stack and to turn on or off the display of the various image channels with the different stainings. The simultaneous visualization of the PolNet interface and the image of each z-section allows us to discriminate and pair each Golgi and nucleus, even in crowded regions. To minimize human error, we suggest that acquisition and polarity delineation are conducted by different persons. To blind the identity of the data set to the person delineating polarity vectors, abstract names should be given to each file.

Accuracy of the flow estimates generated. The accuracy of the WSS estimates produced by the flow solver is of paramount importance for the usefulness of PolNet. Here, we discuss some potential sources of error.

1) Vessel geometry reconstruction: the geometry of the flow domain has a strong influence on the computed hemodynamics. Therefore, it is critical that the process of plexus sample preparation for imaging is done with great care to avoid introducing artifacts (e.g., artificial vessel disconnections) and to ensure that the labeling and mounting protocol does not lead to significant shrinkage of the luminal space. Furthermore, the vessel reconstruction assumes a circular cross section. All these potential sources of error lead to uncertainty in the flow estimates, thus requiring quantification. The user can do this by comparing the flow results against existing experimental measurements of blood velocity in the mouse retinal vasculature (see Bernabeu et al. (14) and Table 2 for values compiled from the literature).
2) Choice of inlet and outlet boundary conditions: the samples under study typically comprise only a subset of the retinal vascular plexus. These take the form of wedges with an artery that runs radially from the optic disc across the wedge and feeds capillary beds located at either side of it, which in turn are drained by veins that return in the same radial fashion to the optic disc (see Franco et al. (5,6)). We term this as the vein-artery-vein (V-A-V) configuration, although other configurations are also possible (e.g., A-V and A-V-A). In addition to imposing no-slip boundary conditions at the vessel walls, we need to specify boundary conditions at the

TABLE 2 WSS Values Reported in the Literature

Reference	Species, Tissue	Vessel	WSS (Pa)
(21)	cat, sartorius muscle	arterioles	6–14
		capillaries	1.2
		venules	0.3–1
(22)	human, various tissues	arteries	1–7
		veins	0.1–0.6
(23)	mouse, infrarenal aorta	artery	8.76 ± 0.83
	rat, infrarenal aorta	artery	7.05 ± 0.67
	human, infrarenal aorta	artery	0.48 ± 0.03

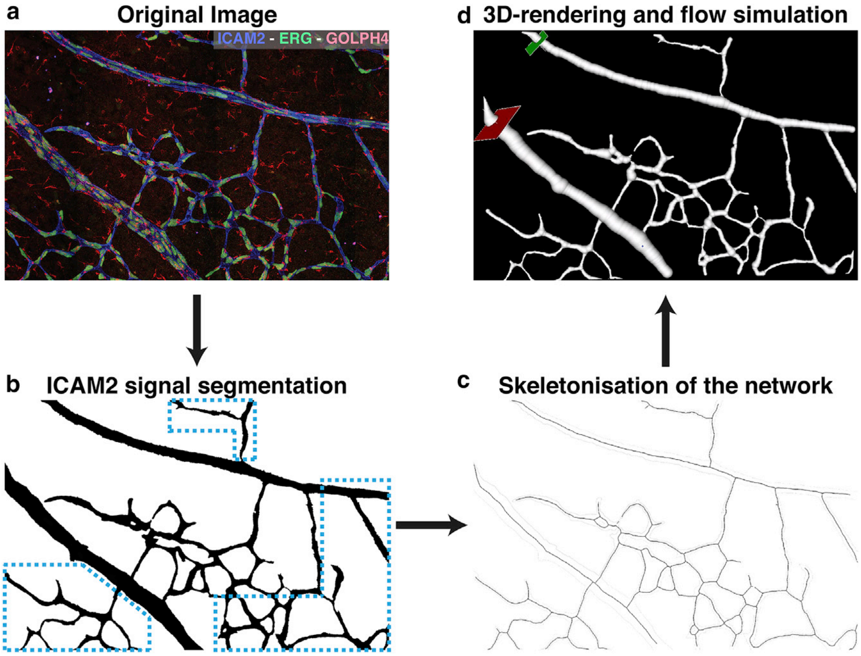

FIGURE 1 (*a*) Example of Golph4 (*red*, Golgi), Erg (*green*, nucleus), and ICAM2 (*blue*, luminal surface) stainings of a subset of a P6 mouse retinal plexus. (*b*) A binary mask generated from the ICAM2 staining in (*a*) is shown. Note that the hemodynamics recovered in the blue highlighted region will not be accurate because of the missing connections to the nearby vessels. The interested reader can refer to (5,6,14) for analyses based on larger plexuses. (*c*) Results of the skeletonisation step are shown. (*d*) A flow simulation setup defines the inlet, outlet, and seed position.

network inlets and outlets. In Bernabeu et al. (14), we surveyed the literature for experimental measurements of blood flow rate or pressure in the central retinal artery/vein to be used as boundary conditions. We concluded that the V-A-V configuration combined with pressure-boundary conditions minimizes the modeling error appearing in the capillary beds defined between each artery-vein pair (including the sprouting front) as well as in the arteries. These are our main regions of interest. Because of the lack of such measurements in the neonatal mouse, we used values measured in adult mice. This design decision can lead to inaccuracies in the flow estimates generated, which are difficult to quantify a priori. In Bernabeu et al. (14), we performed a sensitivity analysis of the inlet/outlet pressures used in our simulations and observed that, although velocity and WSS go up as the pressure difference between inlets and outlets increases, the main perfusion patterns (e.g., areas of relatively low/high flow, flow direction) remain unchanged for a wide range of inlet/outlet configurations and that the relative differences in WSS that have been shown to drive remodeling (5) are also preserved.

3) Choice of rheology model: blood is a dense suspension of deformable cells in blood plasma that leads to a complex range of rheological properties appearing at different scales. When using a CFD approach to simulate blood flow, one has to choose an appropriate approximation for this rheology. In this work, we use the homogeneous shear-thinning rheology model proposed in (14). This approach is a compromise between accuracy and computational tractability (18). However, it fails to capture certain hemorheological features (e.g., the plasma skimming and the Fåhræus-Lindqvist effects) when applied to the simulation of blood flow in small capillaries, especially as vessel caliber gets closer to or even smaller than the typical red blood cell diameter of $\sim 8\mu$m.

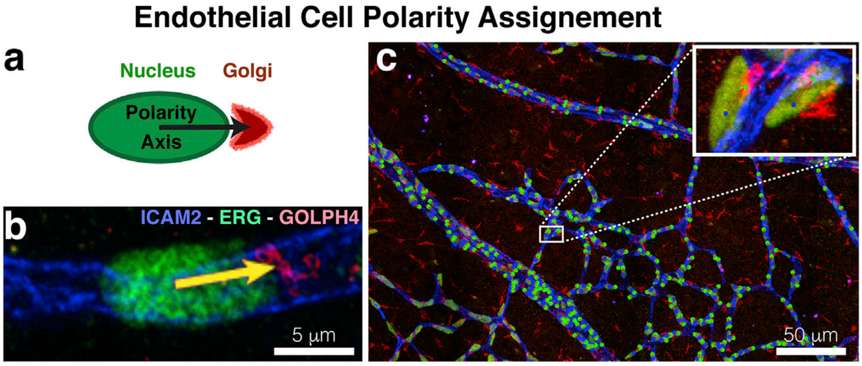

FIGURE 2 (*a*) Polarity vectors are defined between the approximate centers of mass of the nucleus and the Golgi of any given cell. (*b*) An example of a polarity vector on Golph4-Erg-ICAM2 stained plexus is shown. (*c*) A plexus-wide view of the polarity vector delineation on Fig. 1 *a*. A close-in panel provides a detailed view of the polarity delineation on a subset of endothelial cells.

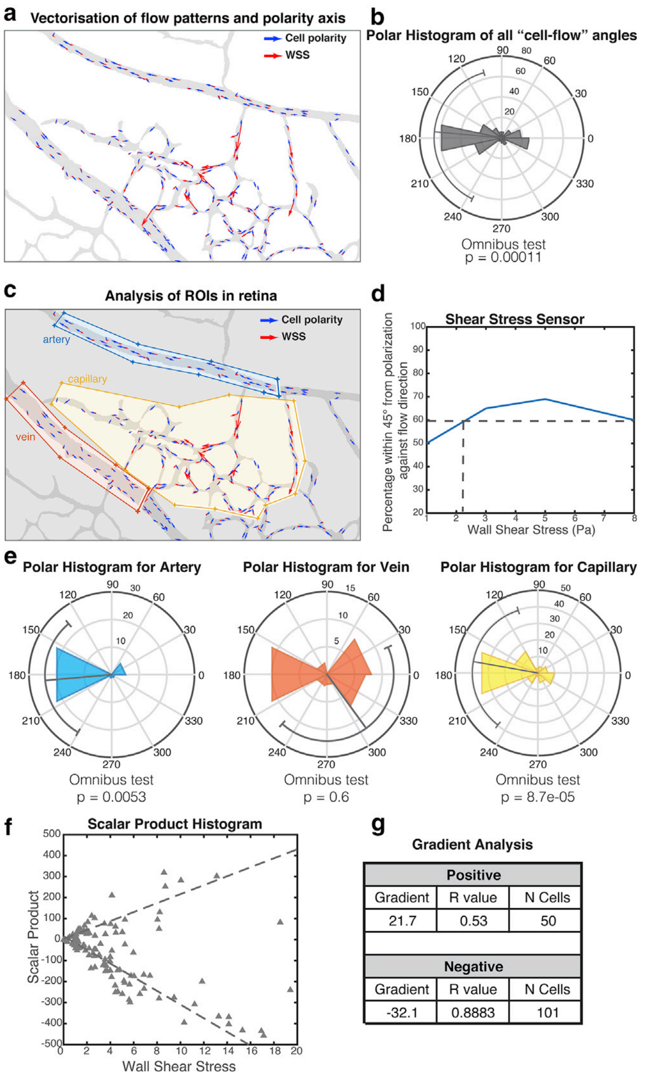

FIGURE 3 Polarity versus wall shear stress (WSS) analysis. (*a*) An overlay of a luminal mask (*gray*), polarity vectors (*blue*), and WSS vectors scaled according to magnitude (*red*) are shown. (*b*) A polar histogram with relative angles formed by the polarity and WSS vectors on each cell are shown, along with the results of a statistical test for random distribution. (*c*) An example of region subdivision is shown: yellow, orange, and blue polygons classify the endothelial cells into arterial, capillar, and venous, respectively. (*d*) Shear stress sensitivity analysis: the percentage of cells polarized against the flow (within 45° tolerance) is shown as a function of the WSS experienced. (*e*) The results of the polarity analysis are applied to each delineated region separately. (*f*) A scatterplot showing the scalar product value for every pair of WSS and polarity vector. (*g*) Scalar product quantification for both the positive and negative subgroups is shown: the gradient of linear regression, Pearson *r*-value, and number of cells.

Results

In this section, we present an example of PolNet analysis performed on the data in Supporting Materials and Methods, Section C, of this article. We choose a subset of a P6 mouse retinal plexus for demonstration purposes. The interested reader can refer to (5,6,14) for analyses based on larger plexuses. Supporting Materials and Methods, Section D, provides troubleshooting advice.

A P6 mouse retinal plexus was prepared, imaged, and postprocessed based on the instructions in Supporting Materials and Methods, Section B, steps 1–20. A subset of interest, including arteries, veins, and capillaries, was identified in the resulting images and cropped to simplify further analysis. Fig. 1 *a* shows the first output image combining the specific stainings (Golph4, Erg, and ICAM2). Fig. 1 *b* shows the second one with the segmentation of the ICAM2 channel.

The ICAM2 mask was processed with PolNet to construct a flow model. Fig. 1 *c* shows the results of the skeletonization step. Fig. S3 *a* presents the vessel caliber histogram generated by the surface reconstruction stage. Fig. 1 *d* shows a screenshot of the flow simulation setup operation (note the location of the flow inlet in *green* and flow outlet in *red*). Finally, Fig. S3 *b* presents the WSS histogram generated at the end of the simulation. Table 2 summarizes experimentally measured WSS values for simulation validation purposes.

The Golph4-Erg-ICAM2 image was used to delineate cell polarity across the whole plexus with PolNet. This is achieved by placing, on top of the image being displayed, consecutive pairs of points corresponding to the approximate center of mass of the nucleus and Golgi of any given cell. Every time a new pair is added, an arrow connecting them is automatically drawn. This arrow defines the polarity vector of a given cell (see Fig. 2, *a* and *b*). Fig. 2 *c* shows the delineation result for all the cells of interest in our example data set.

Fig. 3 presents the results of the PolNet analysis on the example data set. Fig. 3 *a* shows the overlay of the luminal mask and the cell polarity vectors in blue and the flow vectors (WSS selected on the corresponding dropdown menu, not shown) in red. Fig. 3 *b* presents the quantification of the relative angles defined by each pair of polarity and flow vectors. It also includes the results of a statistical test for randomness in the distribution of these angles. Fig. 3 *c* displays an example of subdivision into three regions (arterial, venous, and capillary) for further analysis. Fig. 3 *d* presents the results of the WSS sensor analysis. Finally, Fig. 3 *e* shows the results of the polarity analysis applied to each of the individually selected regions.

Discussion

PolNet was conceived to be an easily extensible tool for network-level quantitative research in vascular morphogenesis. To date, PolNet has proven invaluable for the study of endothelial cell polarization and migration during vascular patterning, as demonstrated by our recent articles (5,6). We have taken special care to make the pipeline easily deployable

across a broad range of computer configurations for easy adoption of the PolNet software by the scientific community. The workflow described in this article is optimized for the quantitative analysis of the relationship between endothelial cell polarity and hemodynamics in the neonatal mouse retina. However, the software can be easily extended to study the relationship between blood flow and other cellular/molecular processes relevant to vascular morphogenesis, including but not restricted to gene expression patterns, changes in endothelial cell morphology, proliferation and apoptosis rates, changes in vessel caliber, or recruitment of mural cells. The only requirement is the ability to image and quantify the signal under investigation and compare it with the spatial distribution of flow parameters computed with the HemeLB CFD solver. This approach was developed for one of the most widely used animal models of angiogenesis: the mouse retina. However, other tissues of interest have more elaborate vascular architectures involving complex 3D vessel configurations. In the future, we plan to extend PolNet to include capabilities to segment and simulate hemodynamics in tissues and organs displaying a highly 3D vascular organization. With the advent of improved clearing techniques (19) and new imaging techniques (20), PolNet will be a powerful analysis method to address the complexity of endothelial cell biology at the network level in intact organs.

SUPPORTING MATERIAL

Supporting Materials and Methods, four figures, and three tables are available at http://www.biophysj.org/biophysj/supplemental/S0006-3495(18)30438-7.

AUTHOR CONTRIBUTIONS

M.O.B., M.L.J., P.V.C., H.G., and C.A.F. designed the research; M.O.B., M.L.J., R.W.N., A.P., and C.A.F. performed the research; R.W.N. and A.P. contributed analytic tools; M.O.B., M.L.J., A.P., and C.A.F. analyzed the data; M.O.B., M.L.J., R.W.N., A.P., P.V.C., H.G., and C.A.F wrote the manuscript.

ACKNOWLEDGMENTS

This work used the ARCHER UK National Supercomputing Service (http://www.archer.ac.uk). The authors acknowledge the contributions of the HemeLB development team and the support of the University College London Research Software Development Group (RSD@UCL) in the completion of this work.

M.L.J.'s work was supported by the Francis Crick Institute, which receives its core funding from Cancer Research UK (FC001999), the UK Medical Research Council (FC001999), and the Wellcome Trust (FC001999). H.G. was supported by a European Research Council consolidator grant called Reshape (311719) and by the British Council's Britain-Israel Research and Academic Exchange initiative. C.A.F. was supported by a European Research Council starting grant (679368), the H2020-Twinning grant (692322), the Fundação para a Ciência e a Tecnologia funding grants (IF/00412/2012, EXPL-BEX-BCM-2258-2013, PRECISE-LISBOA-01-0145-FEDER-016394, and LISBOA-01-0145-FEDER-007391), and a project cofunded by FEDER through Programa Operacional Regional de Lisboa 2020, PORTUGAL 2020, and Fundação para a Ciência e a Tecnologia. M.O.B. and P.V.C. acknowledge support from the UK Engineering and Physical Sciences Research Council under the project "UK Consortium on Mesoscale Engineering Sciences" (grant number EP/L00030X/1). M.O.B., H.G., and C.A.F. acknowledge support by a grant from the Fondation Leducq (17 CVD 03). R.W.N. was funded under the embedded CSE programme of the ARCHER UK National Supercomputing Service (http://www.archer.ac.uk). P.V.C. acknowledges support from the UK Medical Research Council (MR/L016311/1), the UCL Provost, and from European Research Council grants ComPat (671564) and CompBioMed (675451).

REFERENCES

1. Potente, M., H. Gerhardt, and P. Carmeliet. 2011. Basic and therapeutic aspects of angiogenesis. *Cell.* 146:873–887.
2. Chatzizisis, Y. S., M. Jonas, …, P. H. Stone. 2008. Prediction of the localization of high-risk coronary atherosclerotic plaques on the basis of low endothelial shear stress: an intravascular ultrasound and histopathology natural history study. *Circulation.* 117:993–1002.
3. Pries, A. R., and T. W. Secomb. 2008. Modeling structural adaptation of microcirculation. *Microcirculation.* 15:753–754.
4. Hahn, C., and M. A. Schwartz. 2009. Mechanotransduction in vascular physiology and atherogenesis. *Nat. Rev. Mol. Cell Biol.* 10:53–62.
5. Franco, C. A., M. L. Jones, …, H. Gerhardt. 2015. Dynamic endothelial cell rearrangements drive developmental vessel regression. *PLoS Biol.* 13:e1002125.
6. Franco, C. A., M. L. Jones, …, H. Gerhardt. 2016. Non-canonical Wnt signalling modulates the endothelial shear stress flow sensor in vascular remodelling. *eLife.* 5:e07727.
7. Chen, Q., L. Jiang, …, J. L. Du. 2012. Haemodynamics-driven developmental pruning of brain vasculature in zebrafish. *PLoS Biol.* 10:e1001374.
8. Baeyens, N., S. Nicoli, …, M. A. Schwartz. 2015. Vascular remodeling is governed by a VEGFR3-dependent fluid shear stress set point. *eLife.* 4:e04645.
9. Chang, A. H., B. C. Raftrey, …, K. Red-Horse. 2017. DACH1 stimulates shear stress-guided endothelial cell migration and coronary artery growth through the CXCL12–CXCR4 signaling axis. *Genes Dev.* 31:1308–1324.
10. Jin, Y., L. Muhl, …, L. Jakobsson. 2017. Endoglin prevents vascular malformation by regulating flow-induced cell migration and specification through VEGFR2 signalling. *Nat. Cell Biol.* 19:639–652.
11. Poduri, A., A. H. Chang, …, K. Red-Horse. 2017. Endothelial cells respond to the direction of mechanical stimuli through SMAD signaling to regulate coronary artery size. *Development.* 144:3241–3252.
12. Sugden, W. W., R. Meissner, …, A. F. Siekmann. 2017. Endoglin controls blood vessel diameter through endothelial cell shape changes in response to haemodynamic cues. *Nat. Cell Biol.* 19:653–665.
13. Lipowsky, H. H. 2005. Microvascular rheology and hemodynamics. *Microcirculation.* 12:5–15.
14. Bernabeu, M. O., M. L. Jones, …, P. V. Coveney. 2014. Computer simulations reveal complex distribution of haemodynamic forces in a mouse retina model of angiogenesis. *J. R. Soc. Interface.* 11:20140543.
15. Mazzeo, M. D., and P. V. Coveney. 2008. HemeLB: A high performance parallel lattice-Boltzmann code for large scale fluid flow in complex geometries. *Science Direct.* 178:894–914.
16. Benedito, R., S. F. Rocha, …, R. H. Adams. 2012. Notch-dependent VEGFR3 upregulation allows angiogenesis without VEGF-VEGFR2 signalling. *Nature.* 484:110–114.
17. Fantin, A., Q. Schwarz, …, C. Ruhrberg. 2011. The cytoplasmic domain of neuropilin 1 is dispensable for angiogenesis, but promotes

the spatial separation of retinal arteries and veins. *Development.* 138:4185–4191.

18. Gompper, G., and D. A. Fedosov. 2016. Modeling microcirculatory blood flow: current state and future perspectives. *Wiley Interdiscip. Rev. Syst. Biol. Med.* 8:157–168.
19. Richardson, D. S., and J. W. Lichtman. 2015. Clarifying tissue clearing. *Cell.* 162:246–257.
20. Tomer, R., K. Khairy, and P. J. Keller. 2013. Light sheet microscopy in cell biology. *Methods Mol. Biol.* 931:123–137.
21. Popel, A. S., and P. C. Johnson. 2005. Microcirculation and hemorheology. *Annu. Rev. Fluid Mech.* 37:43–69.
22. Chiu, J. J., and S. Chien. 2011. Effects of disturbed flow on vascular endothelium: pathophysiological basis and clinical perspectives. *Physiol. Rev.* 91:327–387.
23. Greve, J. M., A. S. Les, …, C. A. Taylor. 2006. Allometric scaling of wall shear stress from mice to humans: quantification using cine phase-contrast MRI and computational fluid dynamics. *Am. J. Physiol. Heart Circ. Physiol.* 291:H1700–H1708.

Biophysical Journal, Volume *114*

Supplemental Information

PolNet: A Tool to Quantify Network-Level Cell Polarity and Blood Flow in Vascular Remodeling

Miguel O. Bernabeu, Martin L. Jones, Rupert W. Nash, Anna Pezzarossa, Peter V. Coveney, Holger Gerhardt, and Claudio A. Franco

a

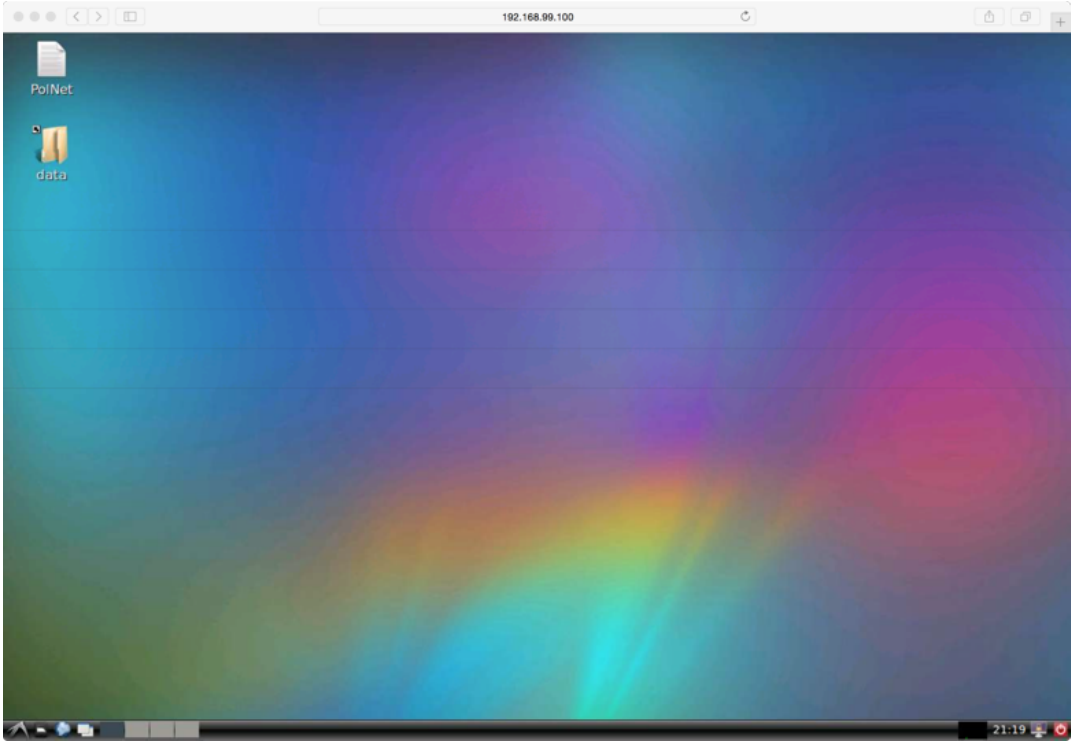

b

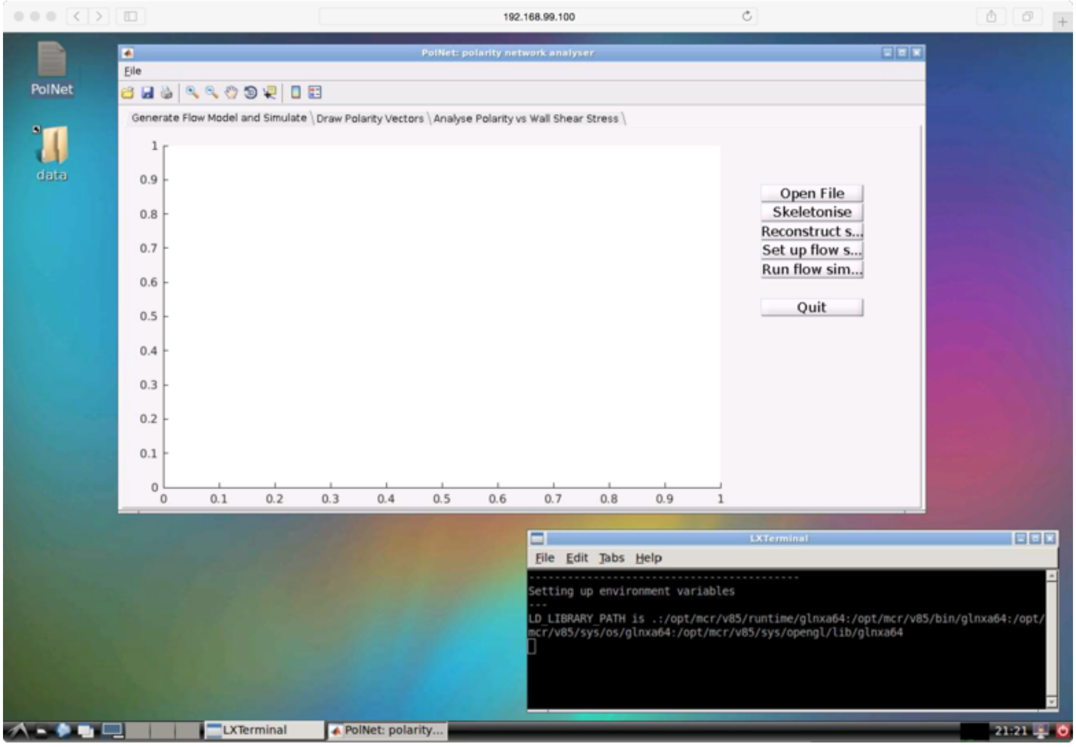

Figure S1: Docker and PolNet installation: a) Web browser displaying the Desktop of the PolNet container, which contains a launcher for the PolNet application and a link to the host data folder mounted on the container file system; b) PolNet application running inside the container, including the main graphical user interface (GUI) window and a console displaying a message log. The different components of the application are organised in three tabs.

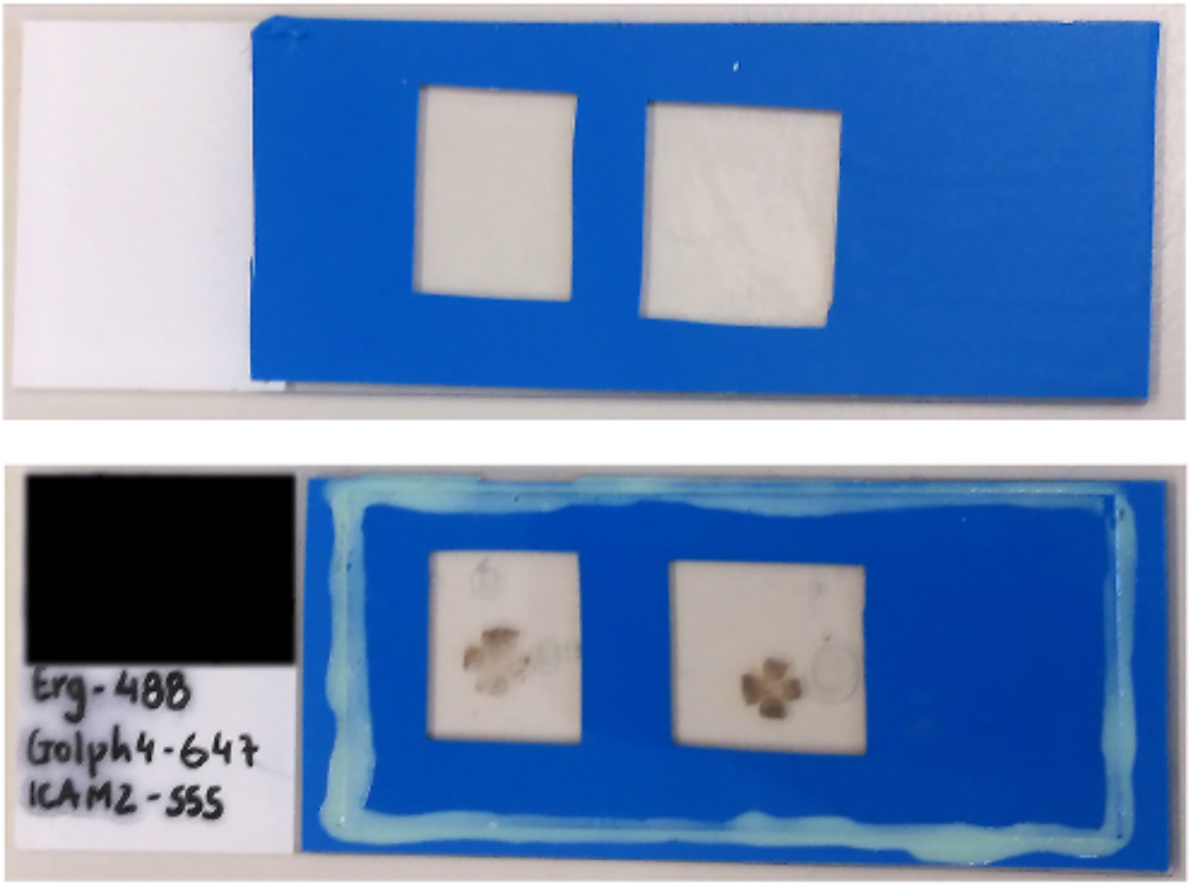

Figure S2: Mounting mouse retinas with minimal 3D squeezing: a) Glass slide pre-processed with microchambers with isolation tape centrally cut with forceps; b) Mounted retinas according to the protocol steps 9-15.

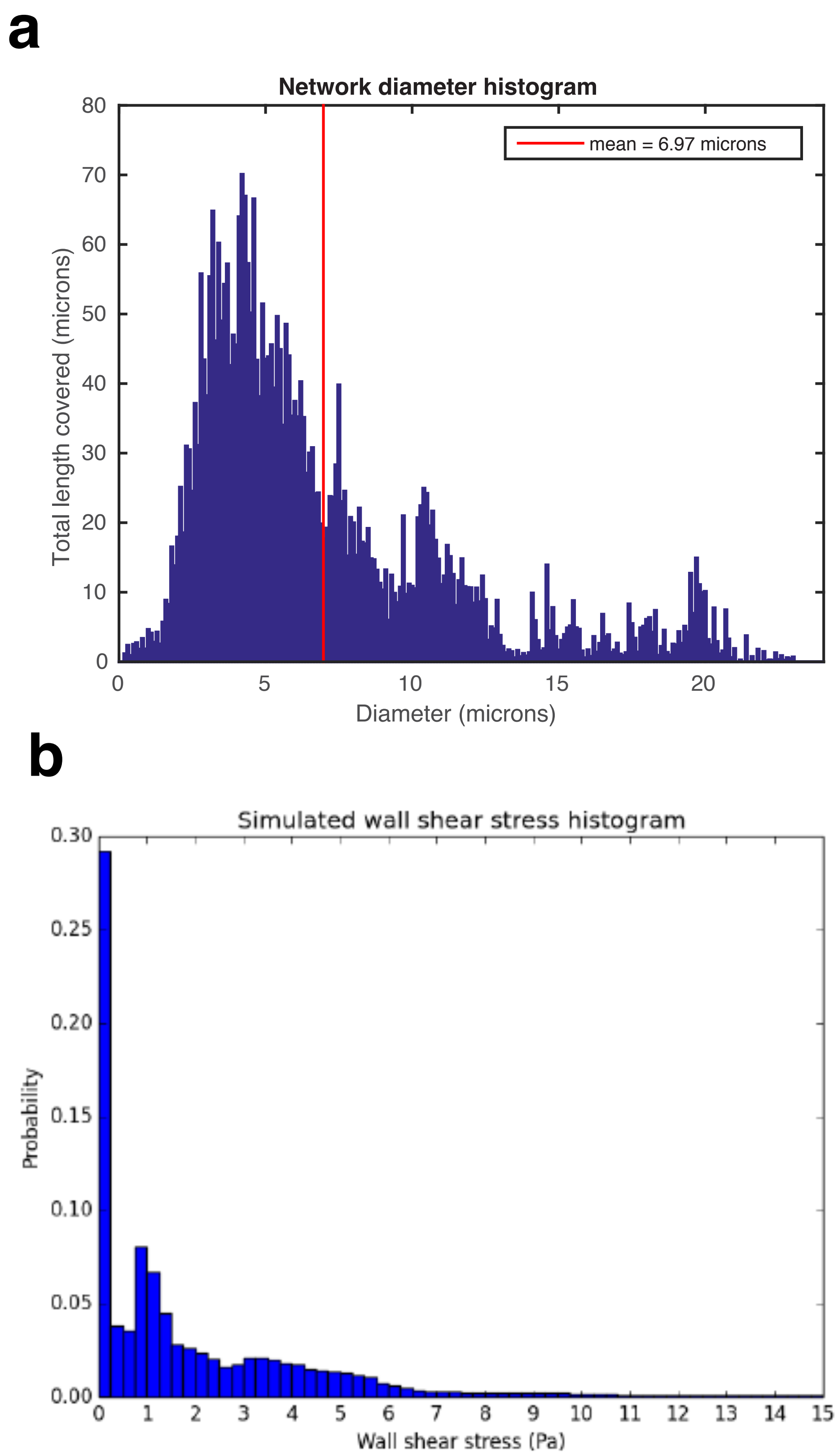

Figure S3: a) Vessel calibre histogram for the computed skeleton. b) Wall shear stress (WSS) histogram. The large proportion of WSS values in the 0-0.25 Pa bin correspond to blind-ended vessels due to recent vessel regression events or those in the region highlighted in Figure 1b.

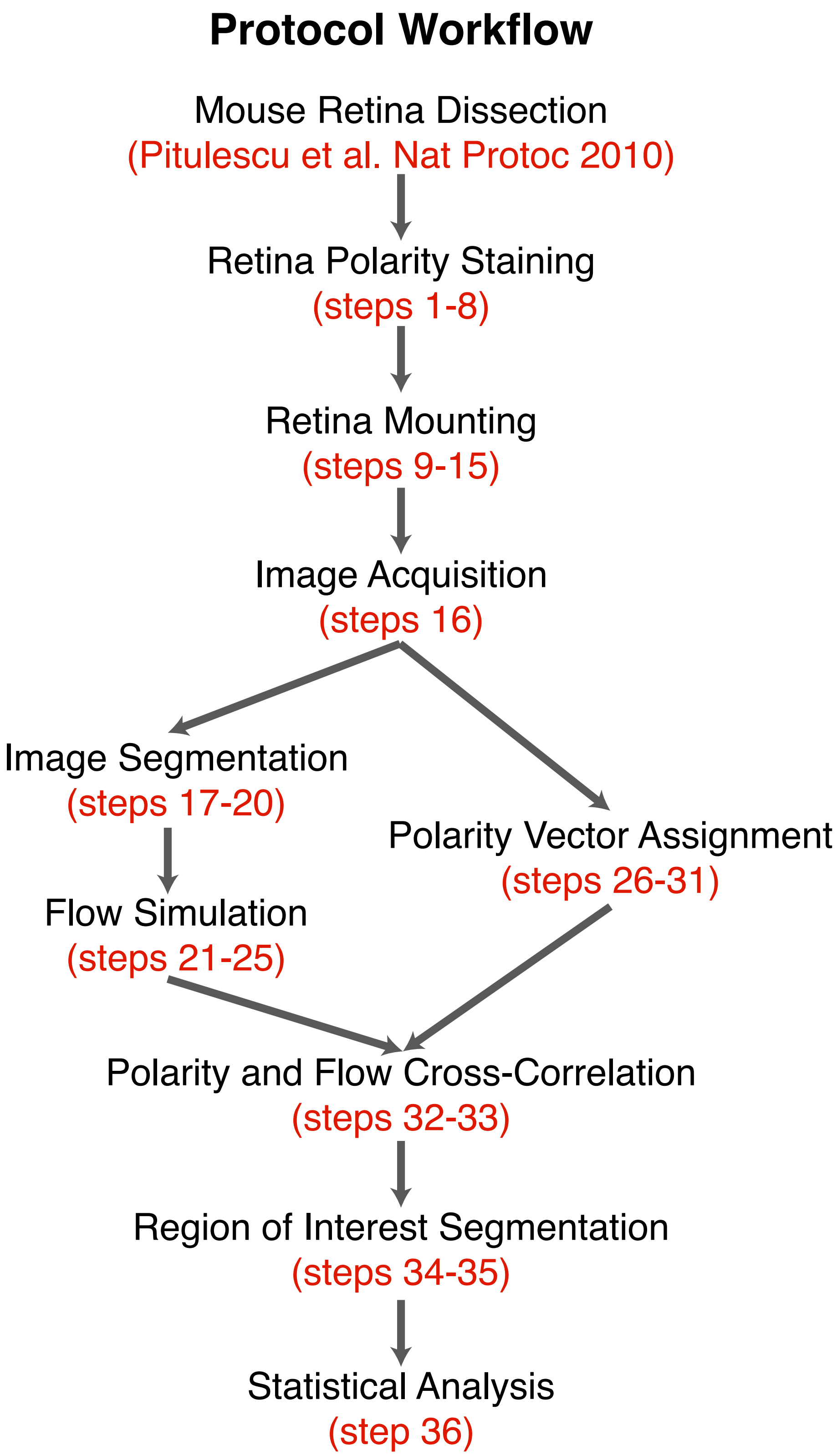

Figure S4: Graphical representation of the protocol workflow. Each of the protocol main stages is listed along with the steps comprising them (between parenthesis). An arrow between two stages indicates a data dependency between the stages at the tip and the base of the arrow.

Table S1: URL to Docker installation instructions for several OSs at the time of writing.

Host's Operating System	Installation instructions
Windows	https://docs.docker.com/docker-for-windows/install/
macOS	https://docs.docker.com/docker-for-mac/install/
Ubuntu	https://docs.docker.com/install/linux/docker-ce/ubuntu/

Table S2: Strings representing paths on the Docker terminal for several OSs. * Note: At the time of writing, Docker only supports mounting host directories within C:\Users (Windows) or /Users (OS X). Blank spaces in the directory names are discouraged.

Operating System	<path_data> substitution example
Windows*	//c/Users/polnet/polnet_data (corresponds to C:\Users\polnet\polnet_data)
OS X*	/Users/polnet/polnet_data
GNU/Linux	/home/polnet/polnet_data

Table S3: Quick reference of PolNet commands and URLs.

Operation	Command/URL	Comment
Launch PolNet container	docker run -i -t -p 6080:80 -v <path_data>:/data mobernabeu/polnet	Substitute <path_data> with the name of the directory containing the data that the user wants to analyse with PolNet. See Table S2 for formats.
PolNet container URL	http://127.0.0.1:6080	
Stop PolNet container	CTRL+C	Use in the terminal from which the container was launched.

SUPPLEMENTARY MATERIAL A: MATERIALS AND SETUP

Reagents
Rat anti-mouse ICAM2 (polyclonal antibody, detects luminal endothelial membrane domains; BD Biosciences, cat. no. 553326)
Mouse anti-rat GM130 (monoclonal antibody, detects Golgi apparatus; BD Biosciences, cat. no. 553326)
Rabbit anti-mouse ERG (polyclonal antibody, detects endothelial cell nuclei; Santa Cruz Biotechnology, cat. no. SC-353)
Alexa Fluor 647 (donkey anti-rat antibody; Invitrogen, cat. no. A21247)
Alexa Fluor 488 (donkey anti-rabbit antibody; Invitrogen, cat. no. A21208)
DAPI [4,6-Diamidino-2-phenylindole dihydrochloride; Sigma-Aldrich, cat. no. D9542]
Vectashield (antifade mounting medium; Vector Laboratories, cat. no. H-1000)
Insulation Tape (AT7 25mm x 33m PVC Electrical Insulation Tape; Advance Tapes, cat. no. 10003626)
Nail polish (any commercially available)
BSA (bovine serum albumin; Sigma-Aldrich, cat. no. A4378-25G)
Sodium Deoxycholate (Sigma-Aldrich, cat. no. D6750) CAUTION! It is harmful if inhaled and swallowed. It is irritating to eyes, respiratory system and skin.
PBS (Dulbecco's PBS, powdered; Biochrom AG, cat. no. L182-10)
FBS (Foetal Bovine Serum; GIBCO cat. no. 10500-064)
Sodium chloride (Carl ROTH, cat. no. 3957.1)
Sodium citrate tribasic dihydrate (Sigma-Aldrich, cat. no. C8532)
Triton X-100 (Sigma-Aldrich, cat. no. T8787) CAUTION! It is harmful if swallowed and can cause serious damage to the eyes.

Equipment
Reaction tubes (1.5, 2, 15, and 50mL)
Laser Scanning Confocal microscope (Zeiss LSM780, Zeiss)
Dissecting instruments: dissection forceps no. 5; short-blade scissors; spring scissors (Fine Science Tools, cat. nos. 91150-20; 14084-08; 15003-08)
Dissection microscope (Zeiss Stemi 305)
Microscope coverslips (Fisher Scientific, Menzel-Gläser, cat. no. 9161028)
Microscope glass slides (Fisher Scientific, Menzel-Gläser, cat. no. 9161145)
Glass Petri dishes
Thermomixer (Eppendorf Thermomixer C). Settings: cycles of 30sec at 400rpm followed by on hold for 5min.
Thermoblock (Eppendorf)
Bitplane Imaris Software / FIJI
Adobe Photoshop software (running on computer with a minimum of 2GB of RAM memory)
Docker software with PolNet container (see Supplementary Material Section B-Timing for suggested computer specifications)

Reagent setup
PBS solution: Dissolve 95.5 g of Dulbecco's PBS powder in 10 liters of ddH2O. Autoclave and store the aliquots at RT for 1 year.
Triton X-100 10% (vol/vol) solution: Dilute 1mL of Triton X-100 in 10mL of PBS 1X. Vortex vigorously until complete homogenisation. Store at 4 °C for 1 year.

Sodium Deoxycholate 5% solution: Dilute 0.5g of Sodium Deoxycholate in 10mL of PBS 1X. Vortex gently until complete homogenisation. Store at 4 °C for 1 year.
PBT solution: Mix 2ml of 10% (vol/vol) Triton X-100 and 98 ml of PBS 1X.
Claudio blocking buffer (CBB): Mix 0.3 g of BSA, 0.1mL FBS, 0.5ml of Triton X-100 10% (vol/vol) solution, 0.1mL of Sodium Deoxycholate 5% solution with 92 ml of PBS 1X. Prepare fresh and store at 4 °C for each experiment.
Claudio antibody buffer (CAB): Mix 25mL of CBB with 25mL of PBS 1X. Prepare fresh and store at 4 °C for each experiment.

Equipment setup

Installing and running PolNet

1 The first step in order to install and run the PolNet application is to install the Docker software in the user's machine, which we will refer to as the host machine in these instructions. Docker allows the execution of so-called containers that emulate a complete operating system and come with pre-installed applications.
Docker's installation process is well documented in the Docker website (https://www.docker.com, at the time of writing) and varies slightly depending on the host machine's OS. For this reason, we point the user to the specific installation instructions maintained by the Docker developers. See Table S1 for the URL corresponding to three of the most widely adopted OSs. Briefly, in a macOS environment, the user should download the installer from the Docker Stable channel (see link in URL provided in Table S1), double-click the downloaded Docker.dmg file to open a new window, and in this window drag the Docker icon to the Applications folder. The interested reader can refer to a detailed Docker tutorial at https://docs.docker.com/get-started/ but this is not strictly required by PolNet users not needing to make changes to the Docker container configuration.

2 In Windows and macOS, launch the Docker application. The first time this is done, the user may be asked to give Docker privileged access to the machine in order to install additional components. A Docker icon will appear in the status bar.

3 Open a terminal and type the following command to launch the PolNet container:
docker run -i -t -p 6080:80 -v <path_data>:/data mobernabeu/polnet
substituting <path_data> with the name of the directory containing the data that the user wants to analyse with PolNet. Table S2 shows some possibilities assuming that the user account name is polnet and that the user has created a directory called polnet_data in his or her home directory. This can be easily adapted to any other user account or directory names.

If this is the first time that the command is run, it will take some additional time to complete since the PolNet container needs to be downloaded.

4 Open a web browser and connect to the address **http://127.0.0.1:6080**. The web browser window will now display the desktop of the PolNet container (see Figure S1a).

5 Double click on the PolNet icon located in the container desktop. This will open two windows: one with the PolNet application Graphical User Interface (GUI) and one

with a console displaying messages associated with the application. The user will interact with the former, the latter functions as an execution log and can be used to diagnose errors in the GUI (see Figure S1b for an example).
Additionally, the user can double click on the data icon in the desktop to check that the directory containing the data to be analysed in the host machine has been correctly mounted in the container.

6 In order to stop the Docker container, the user can close the browser window and then go to the terminal where the container was launched and press **CTRL+C**. If the user wants to restart the container go to Equipment Setup Step 3.
IMPORTANT: The user must store all the important output files in the /data folder of the container (which maps to the host directory specified in Equipment Setup Step 3. Files stored in any other location within the container will be lost when the container is stopped.

SUPPLEMENTARY MATERIAL B: PROCEDURE AND TIMING

Procedure

Figure S4 presents a graphical representation of the protocol workflow with the main protocol stages along with the step numbers comprising them. Arrows between stages indicate data dependencies.

Labelling of the retinal vasculature

1 Collect and fix eyes according to the protocol described in (Pitulescu et al. 2010). However, we recommend a fixation protocol with 2% Paraformaldehyde for 5H at 4 °C in the thermomixer (see Supplementary Material Section A-Equipment for settings) or other means for gentle agitation/mixing. Proceed to dissection of retinas according to (Pitulescu et al. 2010) instructions.

2 Replace the PBS solution with 1mL of CBB to permeabilise/block retinas. Incubate the retinas 2 hours at room temperature in the thermomixer.
CRITICAL STEP CBB is a special buffer that enhances permeabilisation and specificity of antibody binding. This buffer is important to obtain high-contrast fluorescent images for processing.

3 Discard the CBB blocking solution and incubate primary antibodies (anti-Icam2 [dilution 1:200] and anti-Erg [dilution 1:100]) in CAB at 4 °C overnight in the thermomixer. Use 100uL per retina in a round-bottom 2ml eppendorf.

4 Next day, wash retinas 4X 30min with 2mL with PBT at room temperature in a thermomixer.

5 Incubate secondary antibodies (including antiGM130-Alexa555 [dilution 1:800], anti-rabbit Alexa488 [dilution 1:400], and anti-rat Alexa647 [dilution 1:400]) in CAB at 4 °C overnight in the thermomixer. Use 100uL per retina in a round-bottom 2ml eppendorf.

6 Next day, wash retinas 4X 30min with 2mL with PBT at room temperature in a thermomixer.

7 (OPTIONAL) After the final wash, incubate 30min with DAPI 1X solution treatment at this stage, if nuclear staining is used. DAPI staining can help less experienced researchers to distinguish endothelial from non-endothelial Golgi complexes.

8 Wash the retinas once 15min at room temperature in a thermomixer. Proceed to the mounting step.

Mounting mouse retinas

9 Mount the retinas on glass slide, pre-processed with microchambers, as showed in Figure S2a. CRITICAL STEP Microchambers allow enough spacing between the glass slide and the coverslip to allow optimal geometry of the retina. This enables that retinas remain flat-mounted but not squeezed. Squeezing causes vessels to collapse or to distort, which can be problematic for segmentation and flow simulation.

10 Label the glass microscope slides. Transfer both retinas of the same pup to the glass slide using a transfer pipette.

11 Use forceps to position retinas with the inner side containing the vessels facing up (as exemplified in Pitulescu et al. 2010). Carefully remove any debris sticking to the samples. Remove excess PBS solution around the retinas using a 200-µl pipette.

12 Flatten the samples by gently pressing the outer edge of the retinas with the forceps. If necessary, remove more PBS solution from below the edge of the retina. IMPORTANT Retinas must never dry out completely during this procedure.

13 Pipette 60 µl of Vectashield onto a microscope coverslip. Invert the coverslip and place it over the retina. Compress gently to flatten the retinas and spread the mounting media.

14 Absorb any excess of liquid on the edges of the coverslip with a tissue. Seal the edges all around the coverslip with nail polish, as shown in Figure S2b.

15 Keep the slides at room temperature for 1–2 h. They are now ready for imaging or for storage at 4 °C. The best image quality can be obtained within the first 1 week. Retinas, if appropriately mounted, can be stored for up to 6 months.

Retinal vascular network image acquisition and processing

16 Retinas are imaged using a confocal microscope (e.g. Zeiss Laser Scanning Confocal microscope LSM780 with motorized stage or a Zeiss Cell Observer spinning disk microscope). Entire quadrants of the mouse retina are imaged using the tile-scan imaging mode with an oil-immersion 40X objective lens (NA 1.30 at 1,024 × 1,024 pixels). Optimal Nyquist sampling Z-sections are determined directly by the microscope software (e.g. Zeiss ZEN software). Choose regions with no visible damage in the vascular network, and comprising at least the following configuration: artery-vein-artery or vein-artery-vein (this is critical for modelling flow as it provides the inlet and outlet). 3 different channels should be acquired separately: 1) ICAM2 staining; 2) Erg staining; 3) GOLPH4 staining. An additional 4th channel, corresponding to the DAPI staining is also included in case users decided to proceed with Step 7. We would like to highlight that the image that we have included in our protocol (Figure 1a) is just a subset of an entire network. We used this reduced minimalist view to be easier to illustrate the method to readers, although it is not a vessel configuration that should be used in actual experiments. Users should use entire retina plexuses with artery-vein-artery or vein-artery-vein configurations, as shown in Franco et al 2015; Franco et al 2016.

17 Acquired images are processed using FIJI software.

i. Open image using Bio-formats importer: Pluggins>Bio-formats>Bio-formats importer

ii. Create Z-projection: Image>Stacks>Z-project (‘Projection type’: maximum intensity)

iii. Make Composite image: Image>Color>Make Composite

18 The FIJI composite image is then saved as a .tiff (TIFF1).

i. Save image as RGB: Image>Type>RGB; then File>Save

This image will be used to draw axial polarity maps (steps 26-31) of endothelial cells using the PolNet software (Figure 2).

19 In parallel, the layer corresponding to the ICAM2 immunostaining is further processed to create a binary mask to be used to reconstruct the 3D vascular network for the flow simulations (see Figure 1). The workflow involves the following steps:

i. On Composite Image, perform separate Channels: Image>Color>Split Channels

ii. Select ICAM2 channel and discard the other channels.

iii. Threshold ICAM2 signal (Image>Adjust>Threshold): Adjust the threshold so that all the vasculature appears red. Non-vascular dot-like noise is likely to be selected around the image but will be then removed in the next steps. After, selecting the correct threshold, click apply. This will generate a B&W image, where vessels are in black. Make sure that the option “Dark background” in the threshold window in not selected.

iv. To fill small gaps and smoothen the network segmentation and remove noise perform the following three operations:

a. Process>Noise>Remove Outliers; with the following settings: ‘Radius’: 5.0px; ‘Threshold’: 50; ‘Which Outliers’: Dark; then click OK;

b. Process>Binary>Dilate;

c. Process>Binary>Erode.

v. Remove unconnected objects with Analyze>Analyze Particles and the following settings: ‘Size’: 250-Infinity; ‘Circularity’: 0.00-1.00; ‘Show’: Masks; untick all optional boxes. This generates a B&W image of the vascular plexus.

vi. Save the layer as .tiff (TIFF2), taking care of not saving the other layers on the same file.

20 At this step, users should have 2 separate .tiff files, one (TIFF1) containing 3 layers for the specific stainings (Golph4; Erg; and ICAM2); and a second one (TIFF2) containing the segmentation of the ICAM2 staining. These two files should be then copied into the host data directory used by Docker (see Supplementary Material Section A-Equipment Setup for details) to be further processed with PolNet.

Flow model creation and simulation

The following steps are performed in the PolNet software. See Supplementary Material Section A-Equipment Setup for installation and execution instructions. All the functionality in this subsection can be found on the first tab “Generate Flow Model and Simulate”.

21 Open File will present a file selection dialog. Select the black and white mask generated in Step 20 and accept. PolNet will now display the chosen image.

22 Skeletonise will first open a dialog box asking the user to provide the pixel/micrometre ratio of the image. This can be obtained from the imaging setup described in Step 16. Once provided, PolNet will compute the skeleton of the image (and the associated radii of each vessel segment) and overlay it on top of the original image (the original image may be color inverted if it was provided with the opposite

lumen/background tissue color convention) (Figure 1c). The user should inspect the image (use the zoom in/out and pan buttons on the main icon bar) and check that a single skeleton line appears on each vessel segment. If spurious skeleton branches have appeared or the inlet/outlet segments have been shortened in excess go to the Troubleshooting section for instructions on how to tune the configuration of the skeletonisation algorithm.

23 Reconstruct Surface will reconstruct a three-dimensional (3D) model of the vessels luminal surface based on the skeleton and radii computed in the previous step. It will also display a network radii histogram (see Figure S3a) for the user to validate the computed radii against manual measurements obtained directly from the imaged plexus. This step can be time consuming. Because no additional visual output is generated, a message is displayed informing that the operation is running. The user should not try to interact with the application until this message disappears.

24 Setup Flow Simulation will open a new window with the HemeLB Setup Tool and will transfer control to it. In this step, the user will graphically configure the location of the inlets and outlets in the network, provide information about the boundary conditions of the simulation, and generate the discretisation of the luminal space required to simulate flow with HemeLB (Figure 1d). The inlets and outlets are the vessel segments that connect the plexus under study to the rest of the cardiovascular system and are responsible for feeding and draining blood, respectively. The GUI is split into two main parts. The right-hand-side panel is a rendering window that allows the user to zoom, pan, and rotate the 3D model generated in the previous step. The left-hand-side panel presents the main controls used to interact with the application. Most of the information in the left-hand-panel has been prepopulated for the user. The user must specify at least one inlet and one outlet for a simulation to be meaningful as follows:

i. Click on Add Inlet. This will create a new entry in the Inlets and Outlets text box.

ii. Select this new entry and provide an approximate radius for the inlet. This is the radius of a circle inscribed in the square to be displayed. The value can be updated at any time.

iii. Click the Place button and using the mouse to select the location of the inlet region on the 3D model on the right-hand-side window. This is only an initial location that will be fine-tuned in the next step. The user can click on the vessel surface to change the initial location as many times as required. Once the user is satisfied with the location click on the Finish button that will have replaced Place.

iv. Using the right-hand-side window the user can fine tune the location and orientation of the square (note the normal arrow going through the plane. Some of the options are:

a. Drag and drop the plane to move it around. This can be combined with zooming, panning and rotation of the 3D rendered scene to achieve precise positioning.

b. Drag and drop the normal to change the orientation of the plane. The normal vector has arrowheads at both of its ends. The user must ensure that the vector points into the geometry (i.e. an inward facing normal).

c. Drag and drop any of the corners of the square to change the square size.

v. Edit the Pressure text boxes to define the mean pressure at the inlet (first box) and optionally a sinusoidal pressure wave of a given amplitude (second box), phase (third box), and 1 Hz frequency to be added to the mean pressure. In (Bernabeu et al. 2014; Franco et al. 2015), we used a constant inlet pressure of 55 mmHg. See Section Limitations for a discussion on the uncertainty of this choice. The pressure at the outlet will be typically set to 0 mmHg, so that the inlet pressure is equivalent to the total pressure drop across the domain.
Click on the Place button next to Seed position and select a point along the luminal surface contained within any pair of inlet/outlets. A blue sphere will appear, if successful. Click on the Finish button that has appeared where the original Place button was.
Once the previous steps have been carried out, the user can press the Generate button to generate the set of files necessary to run a HemeLB simulation based on the information captured by the GUI. A pop up window will be displayed indicating that the operation is still running. Once the flow model has been generated the user can close the Setup Tool window. The program will then return control to the main PolNet window.

25 Run Flow Simulation will run the flow simulation configured in the previous step. A pop up window will ask the user for the number of CPU cores to be used by the parallel CFD solver. A suggestion is provided based on the number of CPU cores available on the hardware running PolNet (please refer to the Troubleshooting section, if the suggested figure is smaller than the number of physical CPU cores available). Because no additional visual output is generated, a message is displayed informing that the operation is running. After the simulation has completed a pop up window will inform the user whether the simulation has completed successfully or not. See the Troubleshooting section for the most common causes for a simulation to fail. If the simulation terminated successfully, a WSS histogram (see Figure S3b) will be presented to the user to visually validate the results obtained (please see Section Results for some experimentally measured reference values). Note that the flow results are stored in the “results/” subdirectory newly created within the directory containing the original luminal mask used. If a directory with that name already exists, the user will be asked, as a safety measure, whether PolNet should overwrite it or not.

Drawing polarity vectors

The following steps are performed on the PolNet software. See Supplementary Material Section A-Equipment setup for installation and execution instructions. All the functionality in this subsection can be found on the second tab “Draw polarity vectors”.

26 Open File will present a file selection dialog. Select the microscope image showing the nuclei/Golgi markers generated in Step 19 and accept. PolNet will display the image.

27 (Optional) Load Position Data will present a file selection dialog. Select a .csv file with a set of polarity vectors previously saved. This will allow the user to resume a polarity delineation session previously saved.

28 Start Selecting Cells starts a polarity delineation session (or resumes it, if Load Position Data was performed). The mouse pointer will change to a crosshair which allows the user to place, on top of the image being displayed, consecutive pairs of points corresponding to the approximate centre of mass of the nucleus and Golgi of any given cell. Every time a new pair of points is added, an arrow connecting them will be automatically drawn. This arrow defines the polarity vector of a given cell (see Figure 2a and b). The user can press the ESC key at any time to quit the delineation session, which will change the mouse pointer back to its original form. The user can now interact with the application (for instance zooming in/out, panning or using Delete Last Point) and then press Start Selecting Cells in order to resume the delineation session. The final image (Figure 2c) with the positional information of all vectors can be save using the save option of MATLAB.

29 (Optional) The Crosshair Color drop down menu allows the user to change the color of the crosshair in order to enhance contrast against the background image.

30 Save Position Data will present a file selection dialog to save a .csv file with the polarity vectors drawn. This file can be used to: a) resume the delineation session at any time with Load Position Data or b) compare polarity against flow as described in the following section.

31 Delete Last Arrow allows the user to remove the last polarity vector drawn. This operation can be performed multiple times to correct delineation errors.

Polarity vs wall shear stress analysis

The following steps are performed on the PolNet software. See Supplementary Material Section A-Equipment Setup for installation and execution instructions. All the functionality in this subsection can be found on the third tab “Analyse Polarity vs Wall Shear Stress”.

32 Get flow info at cell nuclei will first present a file selection dialog. Select a .csv file with a set of polarity vectors saved in Step 31. PolNet will calculate the value of the flow variable selected in the dropdown menu below (one of wall shear stress, velocity or pressure) at each cell nucleus given in the file. If the user has selected Use last simulation results, PolNet will use the latest simulation results generated in Step 26. Otherwise it will let the user choose a directory containing an existing results subdirectory to calculate the flow at the cell nuclei. This feature is useful if the user needs to analyse multiple delineations (or corrections to an existing one) without having to rerun the flow simulation multiple times.

33 Display mask and vectors will present a file selection dialog for the plexus mask image (.tif extension), immediately followed by a file selection dialog for the file containing the nucleus-Golgi positions (.csv extension). The corresponding flow file is automatically identified and loaded and the vectors corresponding to the nucleus-Golgi positions (blue) and flow magnitude and direction (red) are overlaid onto the plexus mask (Figure 3a). Two plots are generated to give an overview of the dataset: a histogram showing the distribution of scalar product values for all cells and a polar histogram showing the relative angles between the cell and flow vectors (Figure 3b). The distribution of angles is tested for circular uniformity using the Omnibus test

(Berens 2009) under the null hypothesis that the population of angles are uniformly distributed around the circle (i.e. the angles are randomly distributed). The result of the test is displayed in the same figure.

34 Subdivide into regions asks the user for a number of regions to subdivide the image into. The user then draws a closed polygon for each region by left-clicking to set a series of vertices and then either left-clicking on the first point to close the polygon or right-clicking to join the most recent vertex to the first to close the polygon. The cells contained within each polygon are assigned the corresponding label. If regions overlap, the cell belongs to the first region to which it was assigned. The regions can be adjusted by dragging the vertices and extra vertices can be added by holding the “a” key while clicking on an edge. In the case of overlapping regions, the most recently drawn region takes precedence when selecting the vertices/edges. Clicking on the Subdivide into regions button again will restart the process, allowing the user to choose a different number of regions.

35 Analyse Regions will perform the subdivision, colouring the regions according to their grouping whilst preserving the precedence rules for overlapping regions previously described (Figure 3c). A scatter plot is generated showing the flow parameter plotted on the x-axis against the scalar product on the y-axis with the points color-coded according to their region. A series of polar histograms are generated showing the angular distribution between the cell and flow vectors for each region (Figure 3e). If the region polygons are adjusted, clicking Analyse Regions again will redraw the regions to indicate the new groupings and re-generate the series of plots.

36 Save Data opens a file dialog for the user to choose a directory location and filename to store the generated data as a .csv file. This allows the user to perform additional analyses currently not implemented in PolNet App, but important for statistical analysis of samples, including the shear stress sensor analysis (Figure 3d), and scalar product histogram analysis (Figure 3f and g). The output file has a row for each cell and 7 columns, corresponding to: Nucleus x-position; Nucleus y-position; Cell vector length; Flow vector length; Angle between vectors; Scalar Product of vectors; Group. Note that cells that were not including within any of the selected regions are recorded with a Group value of zero. Group are numbered 1 to max number of regions according to the order in which they were selected.

Timing

In this section, we list the most time-consuming tasks in the protocol along with an estimate of the time required to complete them. The tasks not listed here are considered trivial. Many of the computational tasks have a variable duration, which depends on the size and complexity of the vascular plexus being analysed. In those cases, task duration is given in time per unit area of tissue being analysed. To estimate this value we benchmarked PolNet with the example dataset provided with this paper (total area 0.1416 mm2, see Section Results). We benchmarked the code on a MacBook Pro laptop (Apple Inc.) with a 2.5 GHz Intel Core i7 processor, 16 GB of 1600 MHz DDR3 RAM, and a NVIDIA GeForce GT 750M graphics card with 2048 MB of memory. The code was run on a PolNet developer install rather than through a Docker container (see Section Availability and Future Directions for instructions on how to perform a developer install).

Installation

- Download and install Docker: 2-5 minutes depending on internet connection speed.
- Download PolNet container: 3-15 minutes depending on internet connection speed.

Sample preparation, imaging, and postprocessing:

- Labelling of the retinal vasculature: 3 days
- Mounting mouse retinas: 10 minutes per retina
- Retinal vascular network image acquisition and treatment: 4 hours

Flow model creation and simulation:

- Skeletonise: 40 seconds/mm2.
- Reconstruct surface: 13 minutes/mm2.
- Setup flow simulation: 32 minutes/mm2.
- Run flow simulation: 84 hours/mm2 with 4 CPU cores. Additional cores will reduce execution time for as long as a minimum number of flow domain discretisation points per core is ensured. The interested reader can refer to (Groen et al. 2013) for more details about the performance of the flow solver.

Drawing polarity vectors:

- Select cells: 10-15 seconds per endothelial cell nucleus-Golgi pair.

Polarity vs wall shear stress analysis

- Get flow info at cell nuclei: 10 seconds/mm2.
- Analyse Regions: 30 seconds/mm2.

SUPPLEMENTARY MATERIAL C: EXAMPLE DATASET

One example Golph4-Erg-ICAM2 colour image (red-green-blue, respectively).

One example ICAM2 binary image.

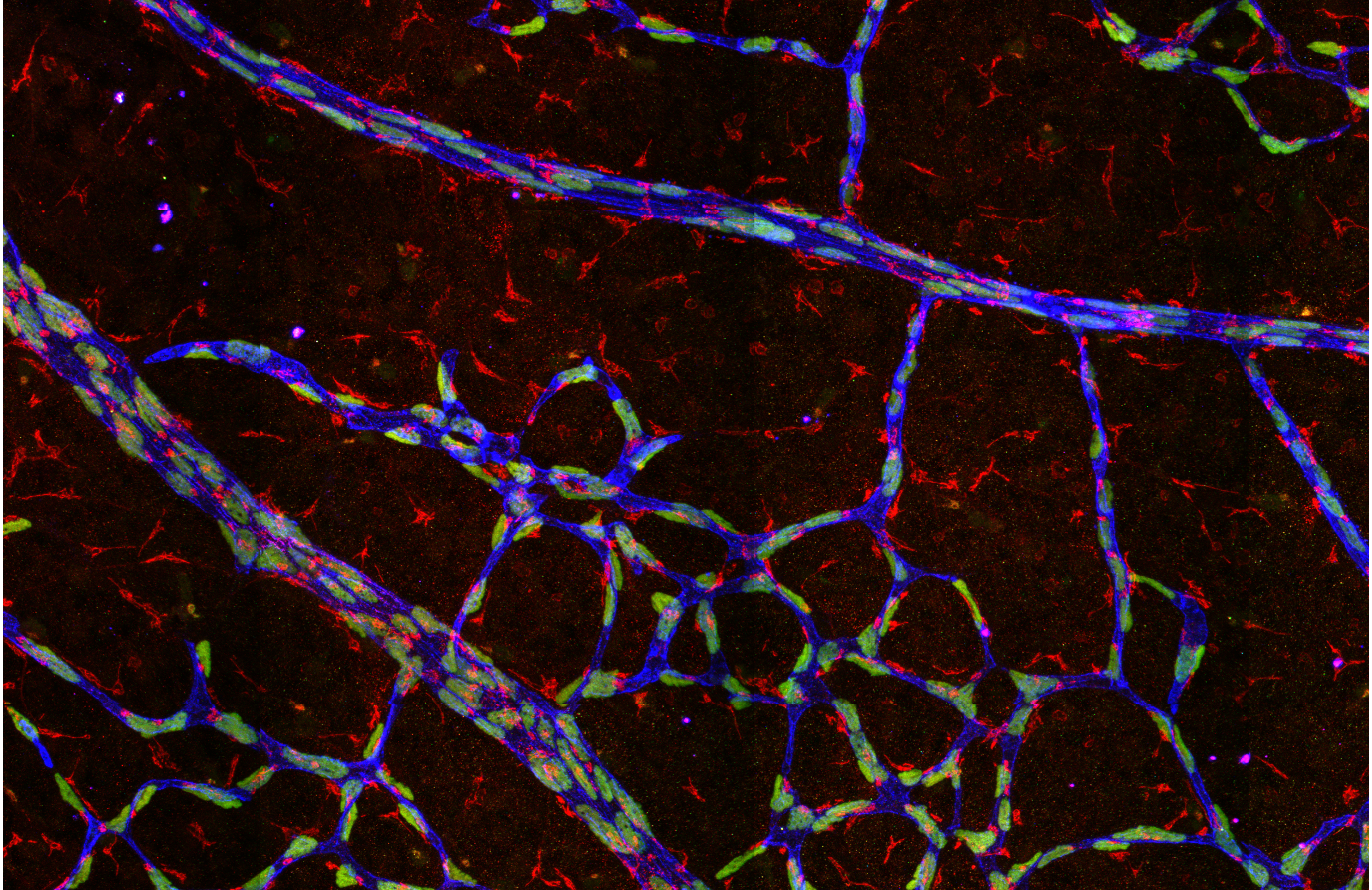

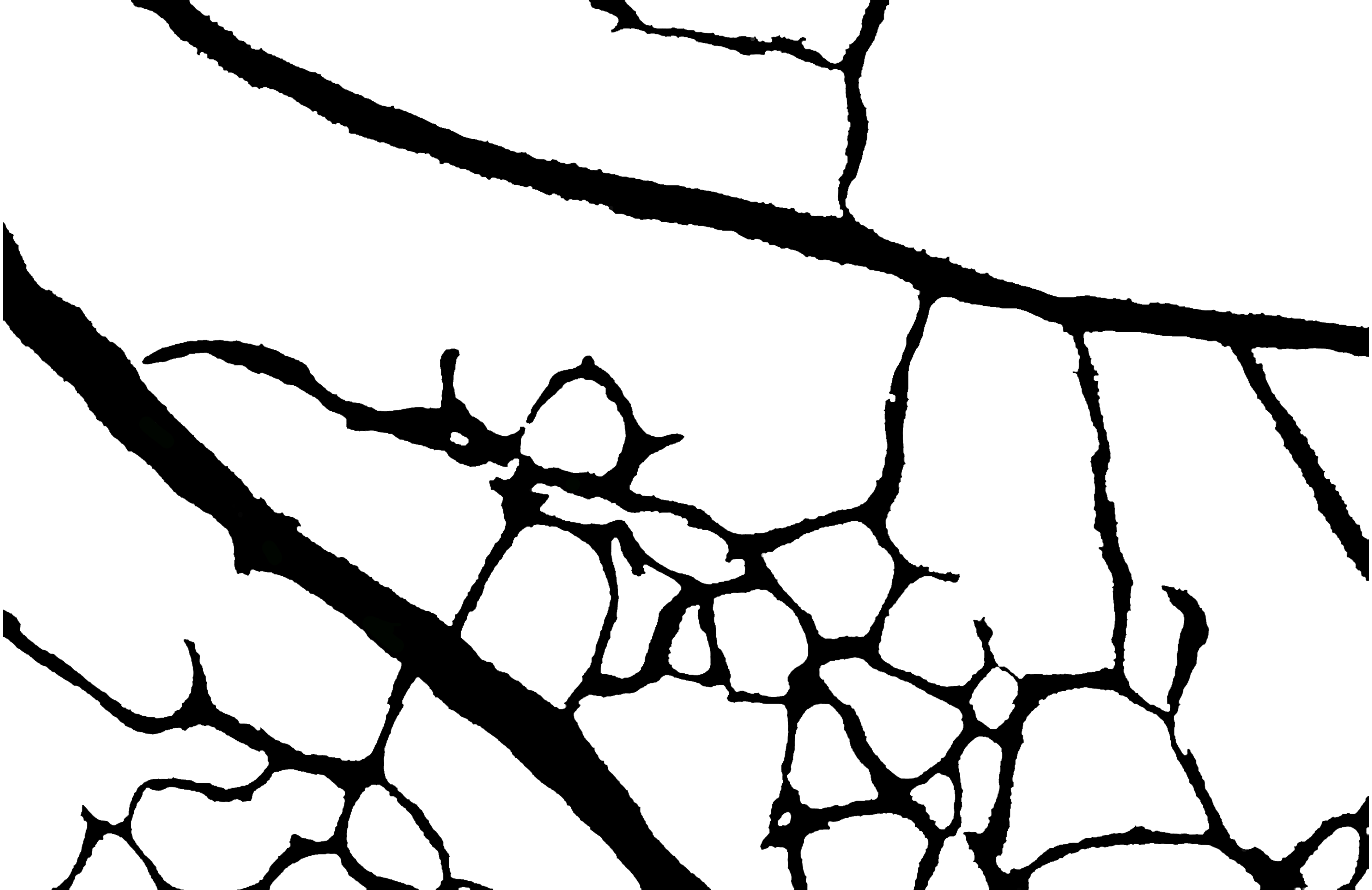

SUPPLEMENTARY MATERIAL D: TROUBLESHOOTING

Problem	Solution	Details
Immunostainings are of poor quality	Verify that all reagents are fresh. Use fresh tissue samples. Refer to (Pitulescu et al. 2010) for additional troubleshooting.	One or more of the immunostainings did not work as expected. Possible issues include, but are not restricted to: no staining; staining with low intensity; cross-reaction between primary or secondary antibodies; non-homogenous staining in the sample.
Plexus reconstruction has missing or disconnected vessels.	Increase the x-, y-, and z-resolution parameters in the configuration window of “Reconstruct surface”. Note than unnecessarily high values will lead to extend execution times for this step.	Depending on the image size and resolution, the “Reconstruct surface” operation may produce a luminal surface model with missing, or artificially narrow, vessels. This can be observed in the “Set up flow simulation” step or by visualising the intermediate .stl file produced during the “Reconstruct surface” step with an external visualiser (e.g. Paraview). This problem arises when the resolution used to sample the network luminal space defined by the image skeleton and radii is not sufficient to resolve all the fine detail in the network.
HemeLB simulation fails due to numerical instability in the flow solver	This issue could indicate an incorrect configuration of the inlets and outlets (e.g. pressure drop is too high). If this is not the case, the user can increase the resolution of the flow domain discretisation by increasing the parameter “Minimum diameter in lattice units” in the configuration window of the “Reconstruct surface” operation.	This problem can be diagnosed if a “Flow simulation failed” message appears after the “Run flow simulation” operation and the PolNet terminal displays the error message “Unstable simulation. Aborting”. This problem is often caused by a choice of flow domain discretisation parameters, which are not sufficiently fine to be able to handle accurately the flow velocities in the domain.
“Reconstruct surface”, “Set up flow simulation” or “Run flow simulation” fail and the PolNet terminal shows a “std::bad_alloc” message.	Configure Docker to have access to a larger amount of memory in the host environment (Docker Preferences, Advanced, Memory) or use a different computer with more available memory.	This problem suggests that PolNet does not have access to enough memory to run one of these stages. This problem arises when the flow model is too big to fit in memory.
